# A “green” strategy to construct non-covalent, stable and bioactive coatings on porous MOF nanoparticles

**DOI:** 10.1038/srep07925

**Published:** 2015-01-21

**Authors:** V. Agostoni, P. Horcajada, M. Noiray, M. Malanga, A. Aykaç, L. Jicsinszky, A. Vargas-Berenguel, N. Semiramoth, S. Daoud-Mahammed, V. Nicolas, C. Martineau, F. Taulelle, J. Vigneron, A. Etcheberry, C. Serre, R. Gref

**Affiliations:** 1 Institut Galien, Université Paris-Sud, UMR CNRS 8612, 92290 Chatenay Malabry, France; 2 Institut Lavoisier, Université de Versailles St Quentin, UMR CNRS 8180, 45 avenue des Etats-Unis, 78035Versailles, France; 3 Cyclolab Cyclodextrin R&D Laboratory Ltd., Budapest, Illatos ut 7, H-1097 Hungary; 4 Department of Chemistry and Physics, University of Almería, Carretera de Sacramento s/n, 04120 Almería, Spain; 5 Plate-forme d'Imagerie Cellulaire, IFR141-IPSIT, Faculté de Pharmacie, 5 rue Jean-Baptiste Clément, F-92296 Châtenay-Malabry, France; 6 Institut de Sciences Moléculaires, Université Paris-Sud, UMR CNRS 8214, 91405 Orsay Cedex, France

## Abstract

Nanoparticles made of metal-organic frameworks (nanoMOFs) attract a growing interest in gas storage, separation, catalysis, sensing and more recently, biomedicine. Achieving stable, versatile coatings on highly porous nanoMOFs without altering their ability to adsorb molecules of interest represents today a major challenge. Here we bring the proof of concept that the outer surface of porous nanoMOFs can be specifically functionalized in a rapid, biofriendly and non-covalent manner, leading to stable and versatile coatings. Cyclodextrin molecules bearing strong iron complexing groups (phosphates) were firmly anchored to the nanoMOFs' surface, within only a few minutes, simply by incubation with aqueous nanoMOF suspensions. The coating procedure did not affect the nanoMOF porosity, crystallinity, adsorption and release abilities. The stable cyclodextrin-based coating was further functionalized with: i) targeting moieties to increase the nanoMOF interaction with specific receptors and ii) poly(ethylene glycol) chains to escape the immune system. These results pave the way towards the design of surface-engineered nanoMOFs of interest for applications in the field of targeted drug delivery, catalysis, separation and sensing.

Metal-organic frameworks (MOFs) are one of the latest classes of ordered porous solids^1^,^2^,^3^,^4^,^5^,^6^. Since their discovery in 1989^7^, MOFs have attracted growing interest due to their useful applications in gas storage, separation, catalysis, sensing^8^,^9^,^10^,^11^,^12^ and, more recently, in biomedicine^13^,^14^,^15^,^16^. One of the key advantages of the MOFs lies in their easily tunable composition. As almost any metal can be associated to polycomplexing linkers (carboxylates, phosphonates, sulfonates, imidazolates…), this has led so far to the discovery of thousands of MOFs^10^,^17^,^18^. Thus, in addition to a huge chemical versatility that can be further enlarged through functionalisation^19^,^20^,^21^, these solids exhibit a large variety in terms of pore sizes and shapes, together with, in some cases, a flexible character which allows to reversibly adapt the MOF pore size to an external stimulus^5^,^22^.

Recently, nanosized MOFs (nanoMOFs) based on porous iron(III) polycarboxylates emerged as a new class of biodegradable and non toxic^23^ nanomaterials of high interest for biomedical applications^13^. NanoMOFs were shown to load unprecedented amounts (within the 20–70 wt% range) of a large variety of therapeutic molecules able to penetrate within the porous MOF structures^13^,^15^,^24^,^25^,^26^,^27^. The important loading abilities of nanoMOFs were related to their high pore volumes and surfaces, the amphiphilic microenvironment inside the pores and the presence of accessible Lewis acidic metal sites. The entrapped drugs were released in a controlled manner in simulated body fluids, while the iron(III) nanoMOFs exhibited intrinsic properties as contrast agents^13^,^23^.

The use of nanotechnology for drug delivery applications is currently bringing a new opportunity to change the landscape of pharmaceutical and biotechnology industries for the foreseeable future, in order to achieve: i) targeted drug delivery; ii) transcytosis of drugs across biological barriers; iii) delivery of drugs to intracellular targets and iv) visualization of sites of drug delivery (theranostics). To reach such challenging goals, it is required not only to achieve high payloads and controlled releases, appropriate sizes and shapes, but also to engineer the shells of the nanoparticles, since the *in vivo* fate of the nanocarriers (biodistribution, pharmacokinetics and targeting abilities) depends upon their surface physicochemical properties^28^.

Whereas several covalent coating methods have been developed in the case of nanocarriers made of mesoporous silica^29^,^30^, so far, there are only scarce examples of surface modified nanoMOFs^13^,^31^,^32^,^33^,^34^,^35^,^36^. Essentially, porous Tb(III)^31^ or Fe(III)^36^ nanoMOFs were covalently grafted with a silica shell, whereas non porous Gd-based materials were covalently modified with a silica shell or with reactive polymers^33^,^34^. However, the complexity of the covalent coating procedures is a drawback for medical applications. Moreover, when degrading, nanoMOFs could release Gd or Tb species with potential toxic side effects. In this context, biocompatible iron-based nanoMOFs appear as better alternatives^13^,^23^, but their coating is a challenge because they are: i) biodegradable, requiring soft grafting methods which ensure the integrity of both the MOF and the drug, and ii) highly porous, which can induce a non-specific adsorption inside the pores^13^ competing with encapsulated drugs.

Therefore, the “ideal” nanoMOFs coating should: i) be obtained by a soft, non-covalent strategy; ii) not penetrate inside the porous structure; iii) not interfere with the entrapped drugs; iv) confer nanoMOF colloidal stability; v) be stable under physiological conditions and vi) be obtained in aqueous media in a single step without using any toxic additive.

To achieve these goals, biodegradable nanoMOFs were synthesis here by a “green” microwave assisted hydrothermal method. The non-toxic mesoporous iron(III) carboxylate MIL-100(Fe) (MIL standing for Material from Institute Lavoisier) nanoMOFs are built up from iron(III) octahedra trimers and trimesate linkers (1,3,5 benzene tricarboxylate) that self-assemble to build a porous architecture delimiting large (29 Å) and small (24 Å) mesoporous cages (Fig.1a). The two types of cages are accessible through microporous pentagonal (5.6 Å) or hexagonal windows (8.6 Å)^37^. Drug loading was typically achieved by soaking the nanoMOFs in solutions of drugs, leading to rapid adsorption of the active molecules within the pores (Fig. 1b). Drugs such as azidothymidine-triphosphate (AZT-TP), the active form of AZT, possessing polar complexing groups, bind eventually the coordinatively unsaturated iron Lewis acid sites (CUS), leading to almost perfect (>99%) encapsulation efficiencies, high payloads and controlled release^24^.

The existence of such strong drug/nanoMOF interactions inspired us to propose an original strategy to coat the nanoMOFs based on the use of aqueous solutions of bulky derivatives that bear strong complexing groups (Fig. 1c). Biocompatible cyclodextrins (CDs) emerged as materials of choice for nanoMOF coating^38^. CDs, well known to improve the physicochemical properties of drugs (stability, solubility and bioavailability)^39^ are considered as “smart” parts of the drug delivery devices^40^. Phosphated CDs (CD-P) were selected since: i) they are bulkier with regard to the nanoMOF windows (15.4 × 7.9 *vs*. 5.6 and 8.6 Å) (Fig. 1c,d) and ii) phosphates are prone to strongly coordinate to the CUS at the nanoMOF surface, thus ensuring coating stability in biological media.

## Results and Discussion

### Physicochemical characterization and stability of the coating

MIL-100(Fe) nanoparticles were incubated with aqueous CD-P solutions and the corresponding rate of grafting CD-P was evaluated by elemental analysis and spectrofluorimetry, using rhodamine-labeled CD-P (CD-P-R; see SI). Remarkably, up to 13, 14 and 17 wt% of CD-P was associated to the nanoMOFs after only 15 min, 1 and 24 hours of incubation, respectively (SI, Tab S1). This indicates a fast kinetic of grafting, with more than 75% of the total coating achieved within less than 15 minutes.

The location of the coatings could be visualized in the particular case of larger MOF particles (>10 μm) which were surface-modified with CD-P-R. Optical sections were observed by confocal microscopy (Fig. 2). The CD-P-R molecules were only detected at the surface (depth compatible with the resolution of the confocal microscope, *i.e.* around 200 nm) of the large crystalline particles. Other examples are given in SI (II.9).

**Figure 1:**
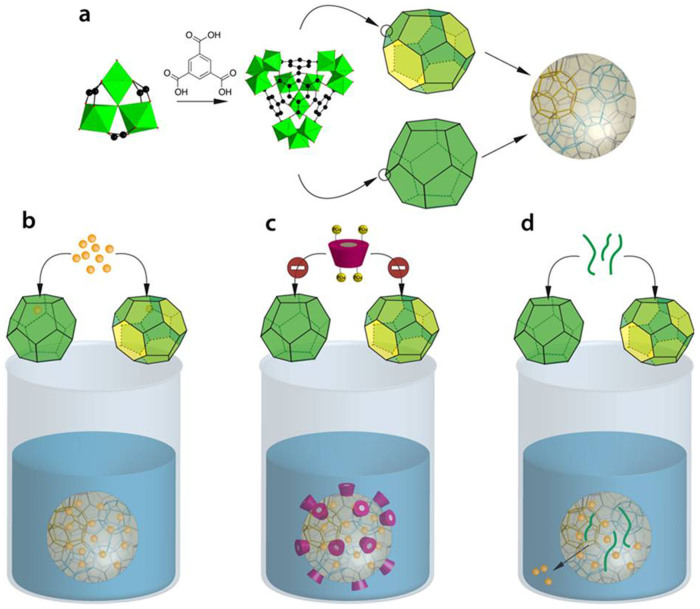
Schematic representation of nanoMOFs “green” synthesis, drug loading and surface modification procedures. (a) NanoMOFs formed by spontaneous coordination between Fe(III) trimers and trimesic acid into hybrid supertetrahedra which further assemble giving rise to a porous zeotypic architecture. Two different types of mesoporous cages delimited by microporous windows are present in this structure: small cages (25 Å) simplified as dodecahedrons with 12 pentagonal faces (openings 5.6 Å) and large cages (29 Å) symbolized as polyhedra consisting of 12 pentagonal and 4 hexagonal faces (openings 8.6 Å). Drug loading can be achieved simply by soaking in aqueous solution. (b) Drug molecules provided with suitable size can be absorbed within the porous core interacting with the nanoparticles matrix by non covalent links with the organic linkers or/and with the Lewis acid Fe(III) sites. (c) Due to their phosphate groups, CD-P strongly interact with the nanoMOFs by coordination with the available Fe(III) sites at the surface, but cannot penetrate inside the matrix because they are too bulky to cross the nanoparticles microporous windows. (d) Coating materials able to penetrate within the MOF porosity can lead to uncontrolled drug release.

**Figure 2:**
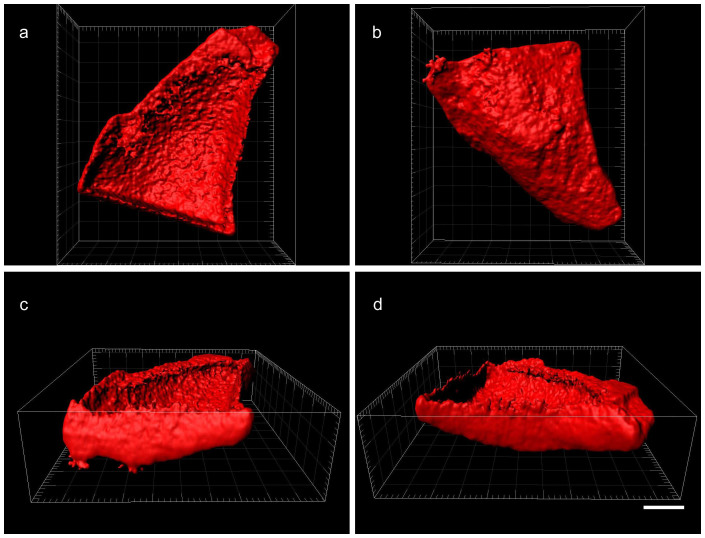
Reconstituted envelope of a MOF crystal coated with rhodamine-labelled CD-P and observed in a confocal microscope. Views from top (a) bottom (b) left (c) and right (d). To enable visualizing inside MOF, signal from the first layer was removed. Bar represents 10 μm.

**Figure 3:**
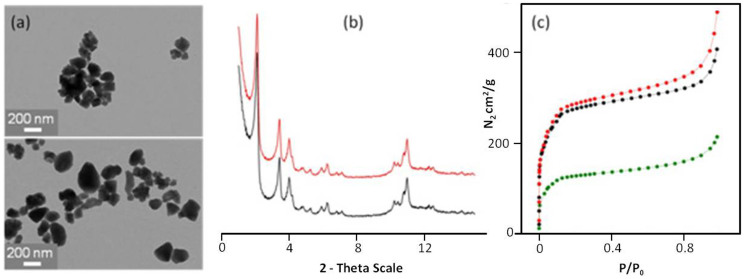
Physico-chemical characterization of the nanoMOFs supramolecular structure and porous surface after modification with CD-P. (a) TEM images of uncoated (upper panel) and coated nanoMOFs (bottom panel) show that the nanoparticles keep the same facetted-type morphology before and after incubation with CD-P aqueous solutions. (b) XRPD patterns of nanoMOFs before (black) and after impregnation with CD-P (red) show that the nanoparticles crystalline structure is not affected by the modification procedure. XRPD patterns correspond to crystalline MIL-100^37^ (c) Nitrogen physisorption isotherms of nanoMOFs (black) and CD-P-modified nanoMOFs (red) were measured by nitrogen absorption at −196°C. The two curves are almost perfectly overlapping, demonstrating that the nanoparticles porous surface is not perturbed after impregnation with CD-P. On the contrary, after incubation with linear PEG chains (green), the nitrogen absorption dramatically decreases, as a consequence of the partial pores occupancy by the polymer chains.

Noteworthy, the CD-P coating on nanoMOFs was stable in aqueous solution, as three extensive washings of the nanoMOFs did not lead to any CD-P leaching (SI, Tab S1). Similarly, three washings with phosphate buffer saline (PBS) lead to only 7% CD-P detachment. In addition, despite the high CD-P content, the surface area of the nanoMOFs was maintained at 1350 ± 100 m^2^.g^−1^ (Langmuir) before and after CD-P association (Fig. 3c). In contrast, although similar coating amounts (17 wt%) were achieved when nanoMOFs were incubated with poly(ethylene glycol) (PEG) aqueous solutions instead of CD-P ones^13^, the surface area dramatically decreased to 350 m^2^.g^−1^, in agreement with a partial filling and/or blocking of the pores by PEG chains. Presumably, PEG chains can penetrate into the pores by reptation (Fig. 1d), their section (~3.1 Å^2^) being smaller compared to the size of the windows^41^,^42^. These studies clearly demonstrate the interest to use coating species with larger rigid sections larger than the windows of the nanoMOFs to avoid pore filling.

The physicochemical characterization of the MIL-100(Fe) nanoMOFs, coated or not, was investigated using a set of complementary techniques (Fig. 3, S1, S2, S3 and S6). First, X-ray powder diffraction (XRPD) and transmission electron microscopy (TEM) studies showed that the nanoMOFs maintained its crystalline structure, size (~200 nm) and morphology (Fig. 3a,b; see also Fig. S3, SI). Dynamic light scattering confirmed that the mean diameters were not affected by incubation with CD-P (224 ± 16 nm and 216 ± 11 nm, respectively).

**Figure 4:**
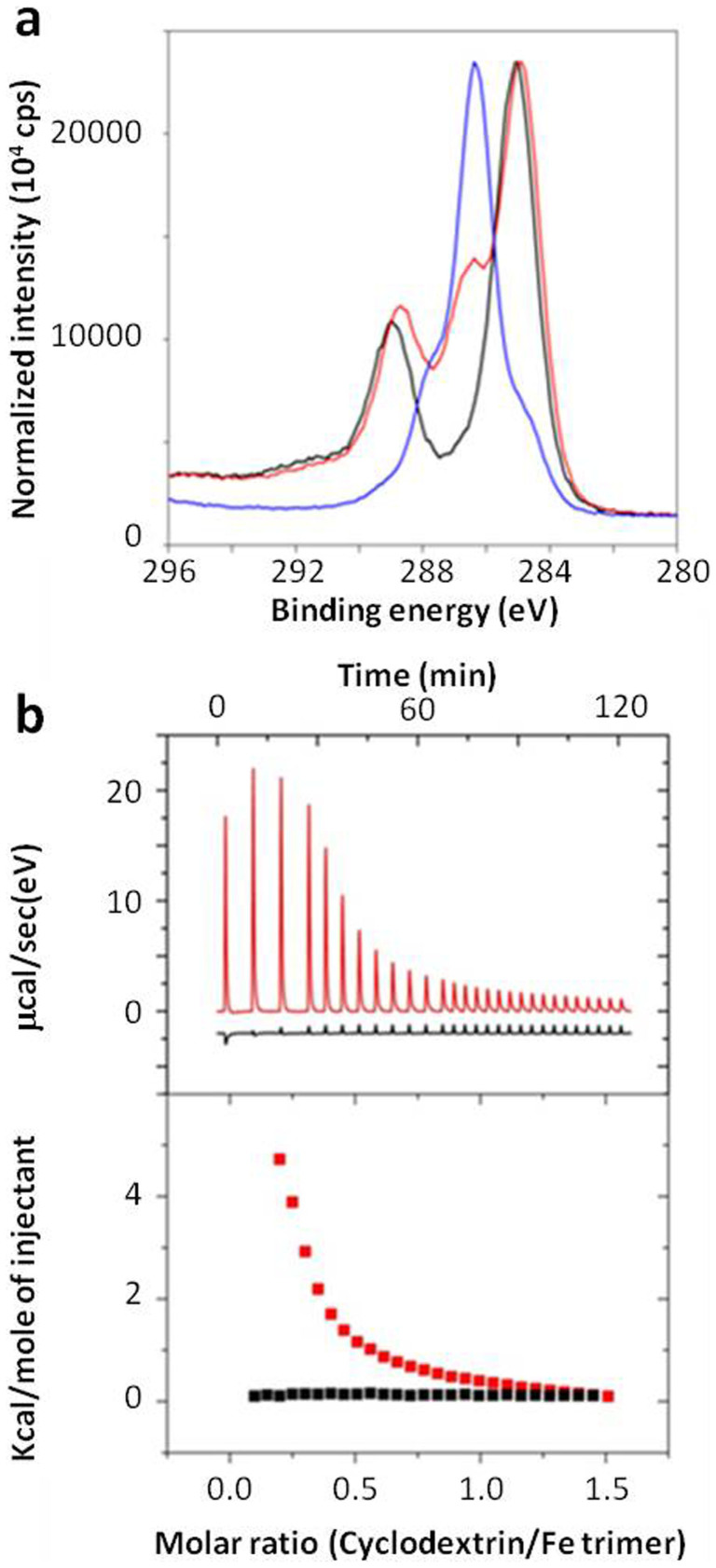
Evaluation of the nanoMOFs-CD-P interaction. (a) C1s binding energy spectra obtained by XPS for nanoMOFs (black), CD-P (blue) and CDP-modified nanoMOFs (red). (b) Thermograms obtained by ITC as the result of the nanoMOF interaction with CD (red) or CD-P aqueous solutions (black).

X ray photoelectron spectroscopy (XPS) was used to ascertain the presence of CD-P within the nanoMOF top layers (~5–10 nm depth) and to determine the quantitative % atomic surface composition. After coating with CD-P, both carbon C1s “fingerprints” of the carbon skeleton of MIL-100(Fe) (284.8 and 289 eV: C-C or C-OOH, respectively) and CD-P “fingerprint” (main contribution at 286.3 eV^43^) were observed (Fig. 4a and Fig S1). The decrease of the % of Fe in the top layers (the C/Fe atomic ratios increased from 7.4 to 12.1 before and after coating, SI, table S2) was indicative of the presence of CD-P in this region. Chemical composition of the top layers corresponds to around one phosphate group per two iron atoms (SI, table S2).

Interestingly, despite the use of a Na salt of CD-P (Na/P ~ 1) to coat the nanoMOFs, their outer surface contained undetectable amounts of Na (SI, table S2). This indicates that the Na^+^/phosphate pair has been replaced by stronger phosphate-iron(III) coordination. Presumably, most of the 3–4 P-O groups *per* CD-P interacted with iron sites, providing thus a cooperative anchoring effect of the CD-P coating. Interestingly, the use of CD without phosphate groups did not lead to any effective grafting (Table S2, Fig. S1).

Isothermal titration calorimetry (ITC) confirmed that the phosphate-iron coordination is the principal binding mechanism. ITC evidenced the absence of interaction between non-phosphated CD and nanoMOFs (Fig. 4b). On the contrary, the binding isotherm for the CD-P/MIL-100 nanoMOF interaction revealed a strong interplay (Fig. 4b) characterized by a ΔG ~ −5.2 kcal.mol^−1^ and ΔH ~ 7.2 kcal.mol^−1^ associated with a prevailing entropic contribution (−TΔS ~ −12.6 kcal.mol^−1^).

Indeed, this is in agreement with the replacement of coordinated water molecules (one *per* iron trimer) inducing a dehydration process characterized by an entropic contribution. Further support of P-O-Fe bonds was given by ^1^H solid-state magic-angle spinning (MAS) nuclear magnetic resonance (NMR) analysis: the^1^H NMR spectrum of the nanoMOF after incubation with CD-P still contains the main features of the empty MIL-100(Fe), indicating that the structure of the MOF was not modified by the incubation (Fig. S3).

**Figure 5:**
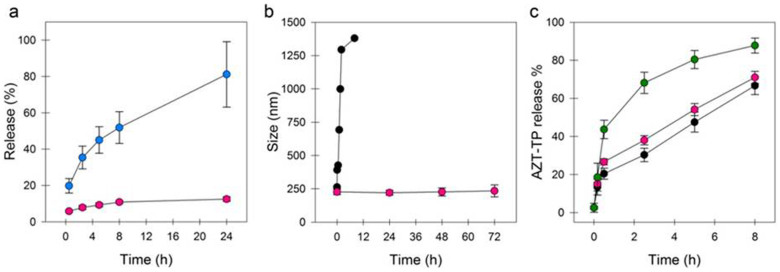
NanoMOFs shell stability under physiological conditions and its effect on the nanoparticles colloidal stability and drug release. a) Kinetics of coating detachment under physiological simulated conditions (PBS 9.5 mM, pH 7.4, 37°C) of rhodamine-labeled βCDP (red) or dextran-biotin-FITC (blue) molecules adsorbed on the nanoMOF surface, measured by fluorescence spectroscopy. b) Water stability of nanoMOFs (black) and βCDP-modified nanoMOFs, measured by DLS. c) AZT-TP release from nanoMOFs (black), βCDP-modified nanoMOFs (red) and PEG-modified nanoMOFs (green) in PBS pH 7.4 at 37°C.

The stability of the coating under physiological simulated conditions was investigated. Indeed, serum is a slightly basic medium (pH ~ 7.4) and contains phosphates that promote the biodegradation of metal carboxylates nanoMOFs^13^. However, whatever the incubation media (phosphate buffer solution (PBS) or cell culture media), less than 10% of the total CD-P coating was detached after 24 hours of incubation (Fig. 5a and S4). It can be concluded that the CD-P-based coating provides a sufficient stability under physiological conditions to play a biological role, knowing that nanoMOFs typically release their drug cargo in less than 24 hours and that nanoparticles generally circulate in the blood stream in shorter time frames^28^. The cooperative effect of the phosphate units of CD-P enables a firm anchorage of the coating, even in media containing competing phosphates. In contrast, in the absence of strong iron complexing groups, as in the case of the previously described dextran-coated nanoMOF (dextran possesses exclusively hydroxyl groups)^13^, a fast and substantial coating detachment was observed in PBS (52 and 81% loss after 4 and 24 h, respectively) (Fig. 5a). In conclusion, not only bulky coating agents, but also strong complexing units are required for stable coatings.

Interestingly, the coating approach could be successfully used for other types of nanoMOFs. For instance, aluminium-trimesate nanoMOFs could be coated by the same way and the resulting CD-P coating was stable (see SI, Fig. S5). Furthermore, 2D NMR investigations confirmed the presence of preferential interactions between the phosphate moieties of CD-P and the Al trimers in the nanoMOFs (Fig S6).

### Effect of the coating on the nanoMOF stability, drug release and cell interaction

As it is the case with other types of uncoated nanoparticles, one of the main drawbacks of uncoated nanoMOFs for their use as drug delivery systems is their poor colloidal stability in aqueous media depending on pH, ions presence and nanoMOF concentration. Fig. 5b clearly shows that uncoated diluted nanoMOFs (200 μg.mL^−1^) underwent a fast aggregation process in water, with mean diameters rising to more than 500 nm within only 30 min. In contrast, CD-P coated nanoMOFs at the same concentration were perfectly stable in water for more than three days, with less than 4% diameter variations. This improved stability could be related to the highly negative surface charge gained by the nanoparticles after their modification with CD-P (ζ ~ −17 ± 3 mV against −35 ± 3.5 mV for uncoated and CD-P-modified MIL-100 nanoMOFs, respectively), providing electrostatic stabilization and thus avoiding aggregation. The ζ-potential values were also kept constant over the same incubation period, in agreement with the coating stability.

Surface-engineering of drug-loaded nanoMOFs was further investigated. Highly hydrophilic anticancer and antiviral drugs such as the phosphate forms of gemcitabin (Gem) or AZT have low affinity for biodegradable polymers and are thus difficult to encapsulate in nanoparticles made using these materials^13^. However, this challenge has been addressed by using MIL-100(Fe) nanoMOFs which could rapidly adsorb AZT-TP by impregnation in aqueous drug solutions (Fig. 1)^24^. AZT-TP has been used as a model drug in this study and has been impregnated in nanoMOFs reaching a loading of 8 wt%. Given its molecular dimensions, AZT-TP is able to cross the hexagonal microporous MIL-100 windows, to strongly interact with CUS located within large mesoporous cages^13^,^24^. After drug loading, nanoMOFs were coated with CD-P by additional one hour incubation with a CD-P aqueous solution. At the end of the incubation, the drug payload was practically unchanged. Indeed, only 2.9 (±1.3)% of the encapsulated drug molecules was lost after surface modification, presumably the drug fraction located near or on the outer surface and which could be displaced by competition with CD-P. Drug release in PBS was progressive and practically the same for coated and uncoated nanoMOFs (Fig. 5c). On the contrary, the AZT-TP loaded nanoMOFs modified with PEG released much faster the drug, with more than 80% after 5 h of incubation (Fig. 5c). This “burst” release could be related to the presence of mobile and hydrated PEG chains inside the interconnected porous structure, which lead to drug expulsion out of the matrix. Therefore, it can be concluded that CD-P coatings do not interfere with drug entrapment and release, in contrast with the deleterious effect of a direct PEG coating of nanoMOFs. In line with these observations, AZT-TP could be loaded into CD-coated nanoMOFs reaching exactly the same loadings (8 wt%) as when first loading the drug and then coating the nanoMOFs. It was concluded that he CD coating does not act as a diffusion barrier for AZT-TP.

**Figure 6:**
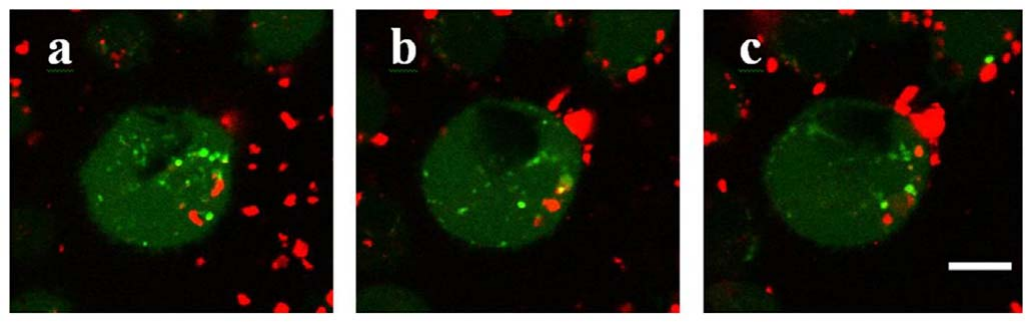
Interaction between J774 macrophages and CD-P coated MOFs studied by confocal microscopy. Cells were stained in green with calcein, whereas the red signal comes from rhodamin-labelled CD-P-coated nanoMOFs. Images represent three distinct optical sections inside cells at different heights above the glass slide: a) 2.3 μm; b) 4.9 μm and c) 7.6 μm. Cell nucleus appears in black. Bar represents 5 μm.

Noteworthy, CD-P coated MIL-100 nanoMOFs were devoid of toxicity, as the uncoated ones (SI, Fig. S7). No significant toxic effects were detected up to high concentrations regardless the type of cells (IC_50_ = 280 μg.mL^−1^ for J774 cell line and >500 μg.mL^−1^ for MCF7 and LP-1 cell lines; SI, Fig. S7). It was previously shown that the CD-P-R coating was stable in the cell culture media (Fig. S4). Confocal microscopy investigations (Fig. 6, particles appear in red) show MOFs particles having penetrated within J774 macrophages with their rhodaminylated coating.

In a nutshell, the one step “green” coating method developed here allows achieving stable coatings even in biological media. It opens up perspectives for a non-covalent functionalization of the MOF surfaces using CD derivatives or CD-based polymers known for their ability to encapsulate drugs^44^. It should be theoretically possible to encapsulate different drugs, in the MOF cores and in the CD shells.

In another approach, a strategy to functionalize the surface of nanoMOFs with PEG has been set up, based on the formation of inclusion complexes between an adamantyl (Ad) endgroup grafted on PEG (PEG-Ad) and β-CD-P cage molecules, shown previously to firmly adhere to the nanoMOF surfaces. Indeed, Ad groups are known to be included and held strongly in β-CD, resulting in high association constants of their derivatives, reaching 10^5^ M^−1^ and excellent stabilities in various media. It is worth pointing out that the first nucleic acid delivery device studied in humans is based on CD-based assemblies coated with PEG-Ad^45^.

In a similar approach, we have used here preformed β-CD-P: Ad-PEG inclusion complexes to coat the nanoMOFs (for details see SI, Fig. S9–S11). These complexes bound strongly to the nanoMOFs, and were firmly anchored, in contrast with PEG chains alone which poorly interacted with the nanoMOFs.

Finally, the CD-Ps on the surface can be chemically modified with ligands. A first proof of concept of ligand grafting has been given here by coating the nanoMOFs' surface with a mannose-bearing CD-P derivative (CD-P-man, for details see SI). Human retinoblastoma cell line Y79, known to overexpress the mannose receptor^46^, has been incubated with uncoated or CD-P-man modified nanoMOF and the nanoparticles uptake has been evaluated by intracellular iron quantification (for details see SI). After exposure to the uncoated nanoparticles, the intracellular iron levels were only slightly higher as compared to those of the untreated cells (Fe ratio = 0.7, for details see SI), indicating the low interactions between nanoMOFs and the Y79 cell line. On the contrary, once functionalized with the CD-P-man, the amounts of nanoparticles able to penetrate inside the cells were more than twice higher (Fe ratio = 1.6 ± 0.4) suggesting an improved nanoMOF uptake likely mediated by the mannose-bearing coating interaction with the mannose receptors on the cellular membrane.

## Conclusion

Our study demonstrates that nanoMOF can be coated with CD derivatives in aqueous media, using a fast, one-step and completely “green” procedure, which do not alter their supramolecular structure and porosity. The coatings, stable in body fluids, can be further functionalized with targeting ligands. These results pave the way for a versatile surface modification of nanoMOFs for targeted multifunctional drug delivery and other applications.

## Methods

### NanoMOF synthesis and characterization

MIL100 iron-trimesate nanoMOFs were synthesized by microwave assisted hydrothermal reaction, heating a mixture containing the iron source (iron(III) chloride hexahydrate) (6.0 mmol) and the organic bridging ligand (1,3,5-benzenetricarboxylic acid) (4.02 mmol), as previously described and detailed in SI section I.1^24^. MIL-100 aluminium nanoMOFs with mean diameters of 120 nm were obtained by microwave-assisted hydrothermal synthesis by heating a mixture of aluminium nitrate nonahydrate (7.0 mmol), 1,3,5-benzenetricarboxylic acid (50 mmol) (for details see SI section I.11). MIL-100 particles of more than 1 μm were synthesized as previously described^37^.

The structure, composition, size and morphology of the resulting nanoparticles were analyzed by X ray powder diffraction (XRPD) and Fourier transform infrared spectroscopy (FT-IR), dynamic light scattering (DLS) and transmission electron microscopy (TEM).

### Synthesis of CD derivatives

CD-P derivatives were synthesized as detailed in SI sections 1–3. Briefly, 6-monoamino-6-monodeoxy-βCD (free base) was obtained by adding 6-monoazido-6-monodeoxy-βCD (11.60 g, 0.01 mol) to a cooled, stirred mixture of 4:1 H_2_O-MeOH (100 mL). Then, a Pd/C suspension (0.58 g, 5% Pd content in 3 mL H_2_O) and hydrazine monohydrate (5 g, 0.1 mol) (5 mL) were sequentially added and the resulting slurry was stirred for 20 min under reflux. The product was purified as white crystals (10.2 g, 90%).

To a stirred solution of 2-azidoethyl 2,3,4,6-tetra-*O*-acetyl-α-D-mannopyranoside (62 mg, 0.148 mmol) in THF (4 mL) was added 2^I^-*O*-propargyl cyclomalto heptaose (174 mg, 0.148 mmol), followed by CuSO_4_ (4.3 mg, 0.029 mmol) and a solution of sodium ascorbate (15 mg, 0.074 mmol) in water (4 mL). The reaction mixture was stirred at room temperature for 18 h and then the solvent was evaporated at reduced pressure. The crude product was purified by column chromatography. 80 mg of this compound (0.053 mmol) were added to a solution of P_2_O_5_ (200 mg, 1.4 mmol) in dried DMF (3 mL). After stirring for 18 h at 40°C, the pH was fixed at 12 with 1 M NaOH and the solution was stirred for 18 h at room temperature. After neutralization (pH ~ 7) with 5% HCl, the CD-P-mannose product was purified by dialysis against water for 5 days and freeze-dried to yield a white solid (143.3 mg, 71%).

### NanoMOF surface modification

The nanoMOFs were surface-modified by impregnation with CD, CD-P, CD-P-R, PEG-amine or dextran-fluorescein-biotin aqueous solutions, under rotative agitation, at room temperature for 15 min up to 24 h. At the end of the incubation the nanoparticles were recovered by centrifugation (10 min at 10000 g) and washed three times with deionized water in order to remove the excess of coating molecules not associated to the surface. Both the pellet, represented by the modified nanoparticles, and the supernatant, containing free coating molecules were collected and analyzed.

The MIL-100 nanoMOF structure and composition before and after modification were evaluated by XRPD, FT-IR, ^1^H MAS Hahn-echo NMR and elemental analysis. The coating localisation was investigated by determining the nanoparticles surface elemental composition using X ray photon spectroscopy (XPS). The porous surface before and after modification with CD-P and PEG-amine was measured by nitrogen sorption experiments at −196°C after samples outgassing at 100°C for 18 h under secondary vacuum.

The thermodynamics of the interaction between CD-P or CD and MIL-100 nanoMOFs was evaluated by isothermal titration calorimetry. Aliquots of 10 μL of CD-P or CD aqueous solutions (13.2 mM) filled into 283 μL syringe were used to titrate an aqueous suspension of MIL-100 nanoMOFs (1.9 mM) into the calorimetric sample cell accurately thermostated at 25°C (SI section I.9). Background of titration consisted on injecting the CD-P or CD aqueous solutions in MilliQ® water.

The stability under physiological simulated conditions (PBS or RPMI supplemented with 10% fetal bovine serum at 37°C) of the fluorescent coatings (CD-P-R and dextran-fluorescein-biotin) was analyzed. After different lapses of time (0.5, 2.5, 5, 24 h) 0.5 mL of supernatant was recovered by centrifugation (10000 × g)/10 min and replaced with the same volume of fresh medium. Released CD-P-R or dextran-fluorescein-biotin was quantified by spectrofluorimetry (SI section I. 10).

The colloidal stability and surface charge of uncoated and CD-P modified MIL-100 nanoMOFs were monitored over 72 h of incubation in water by dynamic light scattering and ζ-potential analysis (SI section I.12).

MIL-100 nanoMOFs were loaded with tritium-labelled azidothymidine triphosphate (AZT-TP). Briefly, 2.5 mg of nanoMOFs were incubated with 500 μL of an AZT-TP aqueous solution 400 μg/mL marked with 1% of AZT-TP[^3^H] (50 μL/3 mL, 3.8 Ci/mmol), 24 h, under rotative agitation, at room temperature. At the end of the impregnation, the nanoparticles were recovered by centrifugation (10000 × g)/10 min. The radioactivity present in the supernatant was determined by scintillation counting and the drug payload (AZT-TP wt%) was calculated according to the formula [1]:


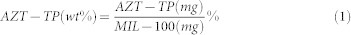
where AZT-TP (mg) is the amount of the entrapped drug in 100 mg of MIL-100 nanoMOFs.

The drug-loaded nanoparticles were coated with CD-P without further purification. For this, 2.5 mg of AZT-TP loaded MIL-100 nanoMOF were incubated with 500 μl of a CD-P aqueous solution 2,5 mg/ml, 500 μl of a PEG-amine aqueous solution 1,67 mg/ml or 500 μl of water as control, 3 h, under stirring at room temperature. At the end of the incubation the nanoMOFs were recovered by centrifugation (10000 × g)/10 min. The supernatant was analyzed by scintillation counting to determine the drug release after surface modification. Finally, the nanoparticles were incubated in PBS at 37°C under rotative agitation. After different incubation times (0.5, 2.5, 5, 8, 24 h) 500 μl of supernatant was recovered after centrifugation (10000 × g)/10 min and replaced with the same amount of fresh medium. The collected supernatants were analyzed by scintillation counting in order to evaluate the AZT-TP release from unmodified, CD-P- or PEG-amine-modified MIL100 nanoMOFs.

The toxicity of the various formulations, coated or not, was assessed by MTT on J774 macrophages, lymphoid (LP1) and breast cancer (MCF-7) cell lines after 48 h of incubation. In addition, the interaction of the nanoMOFs functionalized with CD-P bearing mannose residues was studied on Y79 cell line (human retinoblastoma) overexpressing mannose receptors. The amount of nanoMOFs associated to the cells was determined from the amount of iron associated to the cells. (SI sections I. 14-15).

## Author Contributions

R.G. and V.A. wrote the main manuscript. V.A., R.G., P.H. and C.S. contributed to the nanoMOF synthesis, characterization and surface modification. M.M., A.A., L.J. and A.V.B. synthesized and characterized the CD derivatives. V.A., N.S., V.N. and R.G. performed the cell culture studies and confocal microscopy investigations. C.M., F.T., J.V. and A.E. performed the XPS and NMR investigations. MN and SDM carried on the ITC studies. All authors reviewed the manuscript.

## Supplementary Material

Supplementary Information supplementary information

## Notes

**Dedication:** This paper is dedicated to the memory of Dr. Christian GREF.

## References

[b1] Clearfield A. Metal-phosphonate chemistry. Prog. Inorg. Chem. 47, 371–510 (1998).

[b2] Eddaoudi M. *et al.* Modular chemistry: secondary building units as a basis for the design of highly porous and robust metal−organic carboxylate frameworks. Acc. Chem. Res. 34, 319–330 (2001). 11308306 10.1021/ar000034b

[b3] Chui S. S. Y., Lo S. M. F., Charmant J. P. H., Orpen A. G. & Williams I. D. A Chemically Functionalizable Nanoporous Material [Cu_3_(TMA)_2_(H_2_O)_3_]*n*. . Science 283, 1148–1150 (1999). 10024237 10.1126/science.283.5405.1148

[b4] Férey G. *et al.* A Chromium Terephthalate-Based Solid with Unusually Large Pore Volumes and Surface Area. Science 309, 2040–2042 (2005). 16179475 10.1126/science.1116275

[b5] Kitagawa S., Kitaura R. & Noro S. I. Functional Porous Coordination Polymers**. Angew. Chem. Int. Ed. 43, 2334–2375 (2004). 10.1002/anie.200300610 15114565

[b6] Maspoch D., Ruiz-Molina D. & Veciana J. Old materials with new tricks: multifunctional open-framework materials. Chem. Soc. Rev. 36, 770–818 (2007). 17471401 10.1039/b501600m

[b7] Hoskins B. F. & Robson R. Infinite polymeric frameworks consisting of three dimensionally linked rod-like segments. J. Am. Chem. Soc. 111, 5962–5964 (1989).

[b8] Serre C. *et al.* Role of Solvent-Host Interactions That Lead to Very Large Swelling of Hybrid Frameworks. Science 315, 1828–1831 (2007). 17395825 10.1126/science.1137975

[b9] Yaghi O. M. *et al.* Hydrogen Storage in Microporous Metal-Organic Frameworks. Nature 423, 705–714 (2003). 12750515 10.1126/science.1083440

[b10] Perry J. J., Perman J. A. & Zaworothko M. J. Design and synthesis of metal–organic frameworks using metal–organic polyhedra as supermolecular building blocks. Chem. Soc. Rev. 38, 1400–1417 (2009). 19384444 10.1039/b807086p

[b11] Zhou H.-C., Long J. R. & Yaghi O. M. Introduction to Metal−Organic Frameworks. Chem. Rev. 112, 673–674 (2012). 22280456 10.1021/cr300014x

[b12] Dhakshinamoorthy A. & Garcia H. Catalysis by metal nanoparticles embedded on metal–organic frameworks. Chem. Soc. Rev. 41, 5262–5284 (2012). 22695806 10.1039/c2cs35047e

[b13] Horcajada P. *et al.* Porous metal–organic-framework nanoscale carriers as a potential platform for drug delivery and imaging. Nat. Mater. 9, 172–178 (2010). 20010827 10.1038/nmat2608

[b14] Horcajada P. *et al.* Metal-organic frameworks in biomedicine. Chem. Rev. 112, 1232–1268 (2012). 22168547 10.1021/cr200256v

[b15] Horcajada P. *et al.* Metal-organic frameworks as efficient materials for drug delivery. Angew. Chem. Int. Ed. 45, 5974–5978 (2006). 10.1002/anie.200601878 16897793

[b16] Huxford R. C., Della Rocca J. & Lin W. Metal–organic frameworks as potential drug carriers. Curr. Opin. Chem. Biol. 14, 262–268 (2010). 20071210 10.1016/j.cbpa.2009.12.012 PMC2847625

[b17] Férey G., Mellot-Draznieks C., Serre C. & Millange F. Crystallized frameworks with giant pores: are there limits to the possible? Acc. Chem. Res. 38, 217–225 (2005). 15835868 10.1021/ar040163i

[b18] Yaghi O. Special issue on Reticular Chemistry: Design, Synthesis, Properties and Applications of Metal-Organic Polyhedra and Frameworks. J. Solid State Chem. 178, 2409–2574 (2005).

[b19] Horcajada P. *et al.* How linker's modification controls swelling properties of highly flexible iron(III) dicarboxylates MIL-88. J. Am. Chem. Soc. 133, 17839–17847 (2011). 21950795 10.1021/ja206936e

[b20] Imaz I. *et al.* Metal–biomolecule frameworks (MBioFs). Chem. Commun. 47, 7287–7302 (2011). 10.1039/c1cc11202c 21503346

[b21] Cohen S. M. Postsynthetic methods for the functionalization of metal–organic frameworks. Chem. Rev. 112, 970–1000 (2011). 21916418 10.1021/cr200179u

[b22] Ferey G. & Serre C. Large breathing effects in three-dimensional porous hybrid matter: facts, analyses, rules and consequences. Chem. Soc. Rev. 38, 1380–1399 (2009). 19384443 10.1039/b804302g

[b23] Baati T. *et al.* In depth analysis of the in vivo toxicity of nanoparticles of porous iron(III) metal–organic frameworks. Chem. Sci. 4, 1597–1607 (2013).

[b24] Agostoni V. *et al.* Towards an Improved anti-HIV Activity of NRTI via Metal-Organic Frameworks Nanoparticles. Adv. Healthcare Mater. 2, 1630–1637 (2013). 10.1002/adhm.201200454 23776182

[b25] Chalati T. *et al.* Porous metal organic framework nanoparticles to address the challenges related to busulfan encapsulation. Nanomedicine 6, 1683–1695 (2011). 22122581 10.2217/nnm.11.69

[b26] Cunha D. *et al.* Rationale of Drug Encapsulation and Release from Biocompatible Porous Metal–Organic Frameworks. Chem. Mater. 25, 2767–2776 (2013).

[b27] Horcajada P. *et al.* Flexible porous metal-organic frameworks for a controlled drug delivery. J. Am. Chem. Soc. 130, 6774–6780 (2008). 18454528 10.1021/ja710973k

[b28] Gref R. *et al.* Biodegradable long-circulating polymeric nanospheres. Science 263, 1600–1603 (1994). 8128245 10.1126/science.8128245

[b29] Guerrero-Martinez A., Pérez-Juste J. & Liz-Marzan L. M. Recent progress on silica coating of nanoparticles and related nanomaterials. Adv. Mater. 22, 1182–95 (2010). 20437506 10.1002/adma.200901263

[b30] Lim M. H. & Stein A. Comparative studies of grafting and direct syntheses of inorganic-organic hybrid mesoporous materials. Chem. Mater. 11, 3285–3295 (1999).

[b31] Rieter W. J., Pott K. M., Taylor K. M. L. & Lin W. Nanoscale Coordination Polymers for Platinum-Based Anticancer Drug Delivery. J. Am. Chem. Soc. 130, 11584–11585 (2008). 18686947 10.1021/ja803383k

[b32] Huxford R. C., de Kraft K. E., Boyle W. S., Liu D. & Lin W. Lipid-coated nanoscale coordination polymers for targeted delivery of antifolates to cancer cells. Chem. Sci. 3, 198–204 (2012). 10.1039/C1SC00499A PMC3840543 24288587

[b33] Rowe M. D. *et al.* Tuning the magnetic resonance imaging properties of positive contrast agent nanoparticles by surface modification with RAFT polymers. Langmuir 25, 9487–9499 (2009). 19422256 10.1021/la900730b

[b34] Rowe M. D., Thamm D. H., Kraft S. L. & Boyes S. G. Polymer-modified gadolinium metal-organic framework nanoparticles used as multifunctional nanomedicines for the targeted imaging and treatment of cancer. Biomacromol. 10, 983–993 (2009). 10.1021/bm900043e 19290624

[b35] Taylor K. M. L., Rieter W. J. & Lin W. Nanoscale coordination polymers for platinum-based anticancer drug delivery. J. Am. Chem. Soc. 130, 14358–14359 (2008). 18686947 10.1021/ja803383k

[b36] Taylor-Pashow K. M. L., Rocca J. D., Xie Z., Tran S. & Lin W. Postsynthetic Modifications of Iron-Carboxylate Nanoscale Metal−Organic Frameworks for Imaging and Drug Delivery. J. Am. Chem. Soc. 131, 14261–14263 (2009). 19807179 10.1021/ja906198y PMC2760011

[b37] Horcajada P. *et al.* Synthesis and catalytic properties of MIL-100(Fe), an iron(III) carboxylate with large pores. Chem. Commun. 27, 2820–2822 (2007). 10.1039/b704325b 17609787

[b38] Loftsson T. & Duchêne D. Cyclodextrins and their pharmaceutical applications. Int. J. Pharm. 329, 1–11 (2007). 17137734 10.1016/j.ijpharm.2006.10.044

[b39] Davis M. E. & Brewster M. E. Cyclodextrin-based pharmaceutics: past, present and future. Nat. Rev. Drug Discov. 3, 1023–1035 (2004). 15573101 10.1038/nrd1576

[b40] Gref R. & Duchene D. Cyclodextrins as «smart» components of polymer nanoparticles. J. Drug Del. Sci. Tech. 22, 209–272 (2012).

[b41] Allen C. *et al.* Controlling the physical behavior and biological performance of liposome formulations through use of surface grafted poly(ethylene glycol). Biosci. Rep. 22, 225–250 (2002). 12428902 10.1023/a:1020186505848

[b42] Steels B. M., Koska J. & Haynes C. A. J. Analysis of brush-particle interactions using self-consistent-field theory. Chromatogr. B 743, 41–56 (2000). 10.1016/s0378-4347(00)00206-1 10942271

[b43] Liu Y., Yu Z.-L., Zhang Y.-M., Guo D.-S. & Liu Y.-P. Supramolecular Architectures of β-Cyclodextrin-Modified Chitosan and Pyrene Derivatives Mediated by Carbon Nanotubes and Their DNA Condensation. J. Am. Chem. Soc. 130, 10431–10439 (2008). 18627155 10.1021/ja802465g

[b44] Anand R. *et al.* Citric acid–Y-cyclodextrin crosslinked oligomers as carriers for doxorubicin delivery. Photochem. Photobiol. Sci. 12, 1841–1854 (2013). 23900688 10.1039/c3pp50169h

[b45] Davis M. E. The first targeted delivery of siRNA in humans via a self-assembling, cyclodextrin polymer-based nanoparticle: from concept to clinic. Mol. Pharm. 6, 659–668 (2009). 19267452 10.1021/mp900015y

[b46] Gary-Bobo M. *et al.* Cancer therapy improvement with mesoporous silica nanoparticles combining targeting, drug delivery and PDT. Int. J. Pharm. 432, 99–104 (2012). 22178618 10.1016/j.ijpharm.2011.11.045

