# Supplementary material for: A “green” strategy to construct non-covalent, stable and bioactive coatings on porous MOF nanoparticles
Source: Sci Rep. 2015 Jan 21;5:7925. doi: 10.1038/srep07925 (PMC4300503; doi:10.1038/srep07925)
Supplement: Supplementary Information [file srep07925-s1.doc]

Supplementary Information

A “green” strategy to construct non-covalent, stable

and bioactive coatings on porous MOF nanoparticles

V. Agostoni,^1^ P. Horcajada,^2^ M. Noiray,^1^ M. Malanga,^3^ A. Aykaç,^4^ L. Jicsinszky,^3^ A. Vargas-Berenguel,^4^ N. Semiramoth,^1^ S. Daoud-Mahammed,^1^ V. Nicolas,^5^ C. Martineau,^2^ F. Taulelle,^2^ J. Vigneron,^2^ A. Etcheberry,^2^ C. Serre,^2^ R. Gref.^1*^

**Materials and Methods**

**I.1 Materials**

For the synthesis of 6-monodeoxy-6-mono[(5/6)-rhodaminylthioureido]-β-cyclodextrin phosphate sodium salt: 6-monoazido-6-monodeoxy-β-cyclodextrin is a fine chemical product of CycloLab. Rhodamine B isothiocyanate mixed isomers (RBITC) and hydrazine monohydrate (98%) were purchased from Sigma-Aldrich. Activated palladium/charcoal (10% Pd or 5% Pd content) was from Merck. All the reagents were used without further purification. Solvents were dried by conventional methods and distilled immediately prior to use. Slide-A-Lyzer Dialysis Casette G2, cut-off MW2000 (Thermo Scientific) was used for dialyses. For thin layer chromatography (TLC), silica gel coated aluminium sheets were from Merck (Art. No.: 1.05554). Plates were developed in a saturated chamber in a 10:7 (v/v)1,4-dioxan- 25% aq NH_3_ (v/v) solvent system. Visualization was achieved under UV light at 254 nm and 366 nm, and charring with a solution of 9:1 96% EtOH-96% H_2_SO_4_  followed by heating (105-110 °C). Melting points were determined by a Büchi OP545 and are uncorrected. ^1^H-, ^13^C- and ^31^P-NMR spectra were recorded on a Varian VXR-600 at 400 or 600 MHz. IR spectra were recorded in KBr disk on a Nicolet 205 FTIR.

For the synthesis of β-cyclodextrin phosphate mannose: all reagents were purchased from commercial suppliers and used without further purification unless otherwise noted. TLC was performed on Merck silica gel 60 F_254_ aluminum sheets and developed by UV light and ethanolic sulfuric acid (5 % v/v). Flash column chromatography was performed on Merck silica gel (230-400 mesh, ASTM). Melting points were measured on a Büchi B-450 melting point apparatus and are uncorrected. Optical rotations were recorded on an ADP 220 polarimeter at room temperature. Infrared spectra were obtained as KBR pellets or as films on NaCl plates. ^1^H, ^31^P and ^13^C NMR spectra were recorded on Bruker Avance DPX 300 and 500 MHz spectrometers. NMR Chemical shifts are given in ppm and referenced to internal TMS (*δ*_H_, δ_C_ 0.00). *J* values are given in Hz. ICP-OES results was obtained by using a Termo Scientific iCAP 6500 DUO inductively coupled plasma optical emission spectrophotometer (ICP-OES). Dialysis in water was performed using a 3.500 kDa molecular weight cut-off (MWCO) membrane (Spectra/Por, regenerated cellulose).

Iron chloride hexahydrate (Alfa Aesar, 98%), 1,3,5-benzenetricarboxylic acid ( Aldrich, 95%,) and absolute ethanol (Carlo Erba, 99%) were used for the nanoparticles synthesis and activation. β-CD (Roquette, France), β-CD phosphate (CD-P, phosphated β-cyclodextrin sodium salt, Cyclolab, CY-2017.1, molecular formula: C_42_H_70_O_47_P_4_Na_4_), alpha monomethoxy -omega-amino poly(ethylenglycol) (CH_3_-O-PEG-NH_2_ 5000 Da, Iris Biotech), dextran-fluorescein-biotin (10000 MW, lysine fixable, Molecular Probes) were studied for the nanoparticles surface coating. Phosphate buffer saline (PBS, Dulbecco’s phosphate buffer saline without CaCl_2_ and MgCl_2_, 9.5 mM, Lonza), RPMI (Roswell Park Memorial Institute, basal medium) were used as release media. Azidothymidine triphosphate (AZT-TP, 3'-Azido-2',3'-dideoxythymidine-5'-Triphosphate lithium salt, TriLink) has been loaded within the naoparticles. The radioactive compound AZT-TP-methyl[^3^H] (Azido 3'-deoxythymidine 5'-triphosphate, tetratriethylammonium salt [methyl-^3^H]) was purchased from Moravek.

**I.2 Synthesis of 6-monodeoxy-6-mono[(5/6)-rhodaminylthioureido]-βCD phosphate sodium salt**

(***1***) Synthesis of 6-monoamino-6-monodeoxy-βCD (free base): 6-Monoazido-6-monodeoxy-βCD (11.60 g, 0.01 mol) was added to a cooled, stirred mixture of 4:1 H_2_O-MeOH (100 mL). Then, a Pd/C suspension (0.58 g, 5% Pd content in 3 mL H_2_O) and hydrazine monohydrate (5 g, 0.1 mol) (5 mL) were sequentially added and the resulting slurry was stirred for 20 min under reflux. The reaction mixture was cooled to 50°C, the catalyst was filtered off and washed with water (3x15 mL). After the evaporation of the solvent, the crude product was dissolved in water (50 mL) and 25% aq NH_3_ (2 mL) was added resulting in a white, crystalline precipitate. Crystals were filtered and washed with MeOH (3x10 mL). The solid was dried at 60°C under reduced pressure (10 mbar) overnight in the presence of P_2_O_5_ and KOH yielding 1 as white crystals (10.2 g, 90%). The product was stored in vacuum in the presence of KOH.

(***1***): m.p.: 203-205°C (dec.). *R*_f_: 0.26-0.29.

IR (KBr) ν/cm^-1^: 3428 , 2928, 1080, 1029 .

^1^H-NMR (DMSO-d_6_): δ 5.78–5.63 (m, 14H), 4.90–4.85 (m, 7H), 4.50–4.45 (m, 6H), 3.66–3.54 (m, 28H), 3.42–3.24 ( m, 16H).

^13^C-NMR (75 MHz, DMSO-d_6_): δ 101.8, 82.9, 81.6, 81.5, 73.0, 72.3, 72.1, 59.9.

(***2***) Synthesis of 6-monodeoxy-6-mono[(5/6)-rhodaminylthioureido]-β-CD hydrochloride: To a solution of freshly prepared compound **1** (227 mg, 0.2 mmol) in pyridine (5 mL) was added RBITC (113 mg, 0.21 mmol) and the reaction mixture was kept at 60°C for 4 h. Then, the temperature was increased to 90°-100°C and another portion of RBITC (54 mg, 0.1 mmol) was added. After stirring for 3 h, the reaction mixture was cooled to room temperature. Acetone (20 mL) addition resulted in the obtention of a purple precipitate. The crude product was filtered and washed with acetone (2x1 mL), suspended in water (50 mL) and extracted with water-saturated ethyl acetate (3x50 mL) to remove the unreacted RBITC. The aqueous phase was evaporated at 60°C under reduced pressure and the resulting violet solid was then suspended in water (10 mL), and 0.1 M HCl was added till a clear solution was obtained (pH=4-5). Removal of the solvent by freeze-drying yielded 2 as a violet powder (244 mg, 74%).

(***2***): m.p.: 214-215 °C (dec.). R_f_: 0.52-0.54, 0.82-0.84 (RBITC).

IR (KBr) ν/cm^-1^: 3310 , 2968, 2928 , 1708 , 1617, 1154 , 944 , 683.

^1^H-NMR (D_2_O): δ 0.90-1.30 (m, 12H, RBITC-methyl-H), 3.10-3.50 (m, 14H, H2, H4), 3.50-3.78 (m, 28H, H3, H5, H6), 3.78-4.10 (m, 8H, RBITC-methylene-H), 4.84 (s, 6H, H1), 4.96 (s, 1H, H1’), 6.80-7.00 (m, 6H, aromatic-H), 7.00-7.18 (m, 1H, aromatic-H), 8.00-8.08 (bs, 2H, aromatic-H).

^13^C-NMR (D_2_O): δ 12.25, 45.12, 59.71, 71.83, 72.23, 72.85, 81.31, 95.63, 101.10-101.72 (bs), 112.00-115.00 (bs), 125.40, 129.00-130.93 (bs), 141.40, 154.78, 156.88, 165.40.

(***3***) Synthesis of 6-monodeoxy-6-mono[(5/6)-rhodaminylthioureido]-β-CD phosphate sodium salt: P_2_O_5_ was added (200 mg, 1.4 mmol) to dried *N,N-*dimethylformamide (DMF, 3 mL) and, after the flask was tightly closed, mixture was ultrasonicated till obtaining a clear solution. Then, compound **2** (235 mg, 0.14 mmol) was added and the reaction mixture was stirred at 40°C for 4 h. Then, the mixture t was allowed to cool to room temperature, water (20 mL) was added and the obtained solution was dialyzed for 1 day. The solution was neutralized (pH ~7) with 1 M NaOH and dialysed for 1 day. The pH of the solution was adjusted to 7-8 with 1 M NaOH and then extracted with water-saturatedethyl acetate (4x30 mL). Water was removed under reduced pressure at 60°C till dryness, and the obtained solid was dissolved in water (20 mL), dialyzed overnight and then freeze-dried to yield **3** as a violet powder (229 mg, 84%).

(**3**): m.p.: 229°-232°C (dec.). R_f_: 0.0, 0.82-0.84 (RBITC).

IR (KBr) ν/cm^-1^: 3390 , 1647 , 1594, 1467, 1414, 1348 (P=O), 921, 685, 517.

^31^P-NMR (D_2_O): δ 2.28-1.16, (-)2.78-(-)3.00, (-)10.77-(-)11.28, (-)21.52, (-) 21.78.

**I.3 Synthesis of mannosylated β-cyclodextrin phosphate derivative 4:**

**2^I^-*O*-[1-(2,3,4,6-tetra-*O*-acetyl-α-D-mannopyranosiloxyethyl)-1 *H*-1,2,3-triazol-4-ylmetyl]cyclomaltoheptaose 3:**

2-Azidoethyl 2,3,4,6-tetra-*O*-acetyl-α-D-mannopyranoside (**1**)^1^ and 2^I^-*O*-propargyl cyclomaltoheptaose (**2**)^2^ were synthesized as previously reported. To a stirred solution of **1** (62 mg, 0.148 mmol) in THF (4 mL) was added **2** (174 mg, 0.148 mmol), followed by CuSO_4_ (4.3 mg, 0.029 mmol) and a solution of sodium ascorbate (15 mg, 0.074 mmol) in water (4 mL). The reaction mixture was stirred at room temperature for 18h and then the solvent was evaporated at reduced pressure. The crude product was purified by column chromatography using 10:5:1 CH_3_CN-H_2_O-aq NH_3_ (30%, v/v) as eluant, yielding compound **3** (142.2 mg, 76 %) as a white solid.

mp 213°-215 ºC (dec.).

[α]_D_ 112.3 (*c* 0.2, H_2_O)

IR (KBr): 3390, 2941, 2117, 1750, 1646, 1156, 1081, 1029 cm^-1^;

^1^H NMR (300 MHz, D_2_O): δ 1.80-2.01 (s, 12H, 4CH_3_CO), 3.30-3.50 (m, 18H, H2, H4), 3.50-3.75 (m, 34H, H3, H5, H6), 4.60-4.84 (bs, 6H, H1), 4.96 (s, 1H, H1´), 4.97-5.09 (m, 3H, H2’, H3’, H4’), 8.10-8.20 (bs, 1H, triazol-H)

^13^C-NMR (D_2_O): δ 20.47, 20.59, 20.64, 22.57, 49.03, 60.00, 65.19, 65.77, 67.97, 68.57, 71.85, 72.11, 72.46, 72.76, 73.15, 81.66, 96.39, 102.01, 143.72, 169.66, 170.09

ESI-TOF-MS *m*/*z* calcd for C_61_H_95_N_3_O_45_ 1590.4, found 1590.4 (M + Na)^+^.

**Compound 4**:

P_2_O_5_ (200 mg, 1.4 mmol) was added to dried DMF (3 mL) and the mixture was ultrasonicated, in a tightly-closed flask, until obtaining a clear solution. Compound **3** (80 mg, 0.053 mmol) was added to the solution and the reaction mixture was stirred for 18 h at 40°C. Then, the pH was set to12 using 1M NaOH and the mixture was stirred for 18 h at room temperature. The solution was neutralized (pH ~7) with 5HCl and stirred at room temperature for 1h, dialyzed against water for 5 days and freeze-dried to yield compound **4** as a white solid (143.3 mg, 71%).

mp 224°-226 ºC (dec.).

[α]_D_ 118.6 (*c* 0.2, H_2_O)

IR (KBr) ν/cm^-1^: 3150, 1653, 1504, 1437, 1348 (P=O), 921, 685, cm^-1^

^1^H NMR (300 MHz, D_2_O): δ 3.43-3.51 (m, 18H), 3.69-3.80 (m, 28H), 4.73-4.76 (bs, 7H), 4.92-4.98 (m, 10H), 5.25 (s, 1H), 8.01 (s, 1H, triazol-H)

^13^C-NMR (D_2_O): δ 49.80, 60.77, 62.50, 65.96, 66.55, 68.74, 69.34, 71.87, 72.63, 72.88, 73.24, 73.53, 73.92, 82.43, 96.73, 102.78, 125.45, 144.50, 170.43, 170.86

^31^P-NMR (D_2_O): δ 1.66, 1.15, (-) 8.06, (-) 8.96, (-) 9.67, (-) 9.81, (-) 10.03, (-) 20.90.

ICP-OES: Calc. for C_53_H_67_N_3_Na_20_O_101_P_20_ (3441.3): P, 18.0%. Found: P 18.0%

**I.4 MIL100 Iron-trimesate nanoMOF synthesis and characterization**

MIL100 iron-trimesate nanoMOF was synthesized by microwave assisted hydrothermal reaction as previously described,^3^ heating a mixture of iron(III) chloride hexahydrate (6.0 mmol), 1,3,5-benzenetricarboxylic acid (4.02 mmol) in 20 mL of deionized water, 6 min at 130^o^C under stirring. The power applied was 400 Watt (Mars-5, CEM, US: Power maximum output 1600 ± 240 Watts, Frequency at full power 2450 MHz).

The as-synthesized nanoparticles were recovered by centrifugation 10 min at 10000 g. To remove the residual non reacted organic acid, they were washed in 50 mL of absolute ethanol and recovered by centrifugation 10 min at 10000 g. This activation step was repeated 6 times.

Then, the crystallinity and purity of MIL-100 nanoMOFs were assessed. X ray powder diffraction (XRPD) patterns were collected in a conventional high resolution (θ-2θ) D5000 Bruker diffractometer (λ_Cu_ Kα,Kα_2_) from 3 to 20º (2θ) using a step size of 0.02º and 4° per step in continuous mode. Fourier transform infrared spectroscopic (FT-IR) analyses were performed using a spectrometer (Spectrum two, Perkin Elmer). The nanoparticles size and morphology were characterized by dynamic light scattering (DLS; Malvern® Nano-ZS, Zetasizer Nano series, UK) and transmission electron microscopy (TEM; Darwin; 208 Philips; 60-80-100 KV; Camera AMT). Obtained sizes, crystallinities and Langmuir surfaces were in agreement with previously published data.^3^

The nanoparticles were stored in ethanol at room temperature and further used for *in vitro* assays.

**I.5 MIL-100 nanoMOF surface modification by impregnation with different coating molecules**

MIL-100 nanoMOF was modified by impregnation with a β cyclodextrin (CD), β cyclodextrin phosphate (CD-P), CD-P-R, PEG-amine^4^ or dextran-fluorescein-biotin^4^ aqueous solutions. The incubation was performed under rotative agitation at room temperature for 1h, in the case of CD, CD-P and CD-P-R, 3h in the case of PEG-amine or 24h for dextran-fluorescein-biotin. The initial nanoparticles/coating molecules weight ratio was 1:0.5 (for CD, CD-P, CD-P-R and dextran-fluorescein-biotin) or 1:0.33 (for PEG-amine) and the final nanoparticles concentration was 4 mg/mL (for CD, CD-P and CD-P-R), 2.5 mg/mL (for dextran-fluorescein-biotin) or 15 mg/mL (in the case of PEG-amine).

At the end of the incubation the nanoMOFs were recovered by centrifuging 10 min at 10000 g and washed three times with deionized water (1mL water/ 4 mg of nanoparticles) in order to remove the excess of coating molecules not associated to the nanoparticles surface. Both the pellet, represented by the modified nanoparticles, and the supernatant, containing free coating molecules were collected and analyzed.

The concentration of the fluorescent coating molecules present in the supernatant was determined by emission spectrofluorimetry (CD-P-R: λ_ex_ = 556 nm, λ_em_ = 576 nm; dextran-fluorescein-biotin: λ_ex_ = 488 nm; λ_em_ = 515 nm; Luminescence Spectrometer LS50B, Perkin Elmer). The amount of CD-P-R and dextran-fluorescein-biotin associated to the MIL-100 nanoMOFs was defined by difference.

**I.6** **Physico-chemical characterization of CD-P-modified nanoMOFs**

The MIL-100 nanoMOFs morphology after modification with CD-P was observed by TEM. Uncoated and CD-P-modified MIL-100 nanoMOFs and CD-P, as control, were dried o/n at 100°C and analyzed by FT-IR and ^1^H MAS Hahn-echo NMR. The ^1^H magic-angle spinning (MAS) NMR spectra were recorded on an Avance Bruker 500 spectrometer (B_0_ = 11.7 T), using a 2.5 mm probe. A 90º-180º-90º Hahn-echo pulse sequence was used with 1 µs 90º pulse length. The MAS frequency was 27 kHz, the recycle delay was set to 0.5 s and 1024 transients were accumulated for each sample. The ^1^H chemical shifts were referenced to the proton signal in TMS.

Elemental analysis was carried out on CD-P modified MIL-100 nanoMOFs impregnated with the coating molecules for different incubation times (15 min, 1h, 24h). The nanoparticles were stored at 100°C till the moment of the analysis and their elemental composition (wt% C, O, Fe, P, Na) was determined. The amount of CD-P associated to the MIL100 nanoMOF (βCDP payload) was calculated on the basis of their phosphorous content by the following formula:


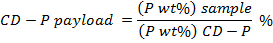


where (P wt%)_CD-P_ is the phosphorous content in CD-P.

**I.7 Nitrogen sorption analyses**

The porous surface of MIL-100 nanoMOFs before and after modification with CD-P and PEG-amine was measured by nitrogen sorption experiments at -196°C on a ASAP 2020 (Micromeritics) after samples outgassing at 100°C for 18 h under secondary vacuum

**I.8 X ray photon spectroscopy (XPS)**

The surface elemental composition was assessed by X ray photon spectroscopy (XPS). Uncoated , CD-P or PEG-amine-modified MIL-100 nanoMOFs and CD-P and PEG-amine aqueous solution were coated on a copper film and analyzed by XPS.

**I.9 Isothermal titration Calorimetry (ITC)**

An isothermal calorimeter (VP-ITC, MicroCal Inc., USA) was used to evaluate the interactions between CD-P or CD and MIL-100 nanoMOFs.

The ITC instrument was calibrated electrically using an internal electric heater, and chemically by measuring the dilution enthalpy of methanol in water. In a typical experiment, aliquots of 10 µL of CD-P or CD aqueous solutions (13.2 mM) filled into 283 µL syringe were used to titrate an aqueous suspension of MIL-100 (1.9 mM) into the calorimetric sample cell accurately thermostated at 25 °C. Intervals between the first three injections were 500 s, then reduced to 300 s (injections 4-12) and finally to 200 till the end of the analysis. Agitation speed was 806 rpm. Background of titration consisted on injecting the CD-P or CD aqueous solutions in solely MilliQ® water placed in the sample cell. The corresponding heat flow was recorded as a function of time accounts for dilution effect.

**I.10 Stability of the fluorescent coatings under physiological simulated conditions**

2 mg of CD-P-R- or dextran-fluorescein-biotin-modified MIL-100 nanoMOFs were incubated with 1 mL of Phosphate Buffer Saline (PBS, pH = 7.4, 9.5 mM), or RPMI supplemented with 10% of fetal bovine serum (FBS) at 37°C under rotative agitation. After different lapses of time (0.5, 2.5, 5, 24 h) 0.5 mL of supernatant was recovered by centrifugation (9491 × g)/10 min and replaced with the same volume of fresh medium. Released CD-P-R or dextran-fluorescein-biotin was quantified by spectrofluorimetry.

**I.11 MIL100-Al Aluminium-trimesate nanoMOF synthesis and characterization**

MIL-100 Al nanoMOFs were obtained by microwave-assisted hydrothermal synthesis heating a mixture of aluminium nitrate nonahydrate (0.007 mol), 1,3,5-benzenetricarboxylic acid (0.05 mol) in 20 mL of deionized water + 4 mL nitric acid 4M, 30 min at 210^o^C with a ramp temperature of 10 min (Mars-5, CEM). The final product was washed in 30 mL of MeOH under magnetic stirrer over night and finally recovered by centrifugation (10000 xg, 10 min). The average hydrodynamic diameter of the as-synthesized MIL-100-Al was 120 nm, as measured by DLS.

**I.12 Water stability of uncoated and CD-P-modified MIL-100 nanoMOF**

Uncoated or CD-P-modified MIL100 nanoMOFs, prepared as previously described, were incubated in water at the final concentration of 200 µg/mL. The nanoparticles size was evaluated after different incubation times (up to 72h) by DLS (Zetasizer Nano 6.12, Malvern Instruments Ltd., UK). The nanoparticles ζ-Potential was also monitored over time (up to 24h, Zetasizer Nano 6.12, Malvern Instruments Ltd., UK). The measurements were performed in a KCl solution 0.5 mM.

**I.13 AZT-TP release from uncoated, βCDP-modified or PEG-amine-modified MIL-100 nanoMOFs under physiological simulated conditions**

MIL-100 nanoMOFs was loaded with a tritium-labelled azidothymidine triphosphate (AZT-TP) aqueous solution, as previously described.^4-5^ nanoMOFs size was unchanged after drug loading.

2.5 mg of nanoparticles were incubated with 500 µL of an AZT-TP aqueous solution 400 µg/mL marked with 1% of AZT-TP[^3^H] (50 µL/3mL, 3.8 Ci/mmol), 24 h, under rotative agitation, at room temperature. At the end of the impregnation, the nanoparticles were recovered by centrifugation (10000 g, 10 minutes). The radioactivity present in the supernatant was determined by scintillation counting using a Beckman Coulter apparatus (LS 6500 multi-purpose scintillation counter) and the drug payload was determined by difference. The drug payloads (AZT-TP wt%) were calculated according to the following formula:

$$AZT-TP (wt\%)=\frac{AZT-TP(mg)}{MIL-100(mg)} \%$$

where AZT-TP (mg) is the amount of the entrapped drug in 100 mg of MIL-100 nanoMOFs.

2.5 mg of AZT-TP loaded MIL-100 nanoMOF were incubated with 500 µl of a CD-P aqueous solution 2,5 mg/ml, 500 µl of a PEG-amine aqueous solution 1,67 mg/ml or 500µl of water as control, 3h, under stirring at room temperature. At the end of the incubation the nanoparticles were recovered by centrifuging 10min at 10000 g. The supernatant was analyzed by scintillation counting to determine the drug release after surface modification. Finally, the nanoparticles were incubated in 1ml of PBS at 37°C under rotative agitation. After different incubation times (0.5, 2.5, 5, 8, 24 h) 500µl of supernatant was recovered after centrifugation (10 min at 10000 g) and replaced with the same amount of fresh medium. The collected supernatants were analyzed by scintillation counting in order to evaluate the AZT-TP release from unmodified, CD-P- or PEG-amine-modified MIL100 nanoMOFs.

**I.14 Quantification of iron levels within Y79 cell line by iron staining**

Y79 cell line (human retinoblastoma) was grown in RMPI 1640 supplemented with 20% (v/v) heat-inactivated fetal bovine serum. Cells were maintained in a humidified incubator with 95% air/5% CO_2_ at 37 °C.

1.5x10^6^ cells were plated in a 24 wells plates. After 24h they were incubated 24h with 1 mL of RPMI 1640 20 % FBS or 1 mL of culture media containing uncoated or CD-P-mannose modified nanoMOFs (nanoMOF concentration = 100 µg/mL). At the end of the incubation, the samples were collected and filtered (Millipore, Isopore^TM^ membrane filters, pore size 3µm). Cells recovered on the filter were incubated with 100 µL HCl (5M) at 60°C for 2h. At the end of the incubation, 100µL of 4% potassium ferrocyanide was added in each well. The plates were further incubated at room temperature in the dark for 30 min. The absorbance of the treated samples was measured at 700 nm and compared to that of the untreated cells.

**I.15 Confocal microscopy investigations**

Live cell imaging and particles observations were carried on with an inverted confocal laser scanning microscope LSM 510-Meta (Carl Zeiss, Germany) using a Plan-Apochromat 63X/1.4 objective lens, equipped with an argon (488 nm excitation wavelength) and a helium neon laser (543 nm excitation wavelength). Calcein and rhodamine fluorescence were collected with a 505-550 nm band-pass and a 560 nm long pass emission filter respectively, under a sequential mode. The pinhole was set at 1.0 Airy unit. 12 bit numerical images were acquired with LSM 510 software version 3.2. The 3D reconstructions and surface rendering were performed with Imaris® software v.7.1.1 (Bitplane Scientific Software, St. Paul, MN).

J774 cells were cultured in 6 wells plates containing round coverslips, 25 mm diameter (Fisher Scientific). Just before the confocal experiments, coverslips were removed and mounted in an Attofluor® Cell Chamber (A-7816), Invitrogen. One mL cell culture medium (DMEM, 10% SVF) containing calcein was added and the cells were incubated for 20 min. The medium was withdrawn, the cells were washed thrice and then incubated with 1 mL cell culture media containing 1 mg large (> 10 μm) MOF crystals coated with rhodaminylated CD-P. These objects were also observed separately, in the absence of cells, to study their coating.

**Supplementary References**

1. A. L. Martin, B. Li and E. R. Gillies, *J. Am. Chem. Soc.*, 2008, **131**, 734-741.

2. J. M. Casas-Solvas and A. Vargas-Berenguel, *Tetrahedron Lett.*, 2008, **49**, 6778-6780.

3. V. Agostoni, P. Horcajada, V. Rodriguez-Ruiz, H. Willaime, P. Couvreur, C. Serre and R. Gref, *Green Materials*, 2013, DOI 10.1680/gmat.13.00001.

4. P. Horcajada, T. Chalati, C. Serre, B. Gillet, C. Sebrie, T. Baati, J. F. Eubank, D. Heurtaux, P. Clayette, C. Kreuz, J.-S. Chang, Y. K. Hwang, V. Marsaud, P.-N. Bories, L. Cynober, S. Gil, G. Ferey, P. Couvreur and R. Gref, *Nat Mater*, 2010, **9**, 172-178.

5. V. Agostoni, T. Chalati, P. Horcajada, H. Willaime, R. Anand, N. Semiramoth, T. Baati, S. Hall, G. Maurin, H. Chacun, K. Bouchemal, C. Martineau, F. Taulelle, P. Couvreur, C. Rogez-Kreuz, P. Clayette, S. Monti, C. Serre and R. Gref, *Adv. Healthcare Mater.*, 2013, 2(12), 1630–1637.

**Supplementary Results**

**II.1 MIL-100 nanoMOFs modification with CD-P: kinetics of interaction and payload associated to the nanoparticles**

MIL- 100 nanoMOFs were modified simply by impregnation into a CD-P aqueous solution. At the end of the incubation the nanoparticles were recovered by centrifugation, washed with water in order to remove the excess of free molecules not associated to the matrix and finally characterized.

The kinetics and the amount of CD-P associated to the nanoparticles were characterized by elemental analysis. The CD-P payload interacting with the nanoparticles was found to be: 13.1, 14.1 and 17.3 wt% after 15 min, 1h and 24h of incubation, respectively (Tab. 1). These results suggested a fast kinetics of CD-P adsorption on the nanoparticles since almost all the interacting molecules were already bound to the matrix after only few minutes of impregnation. Moreover, after three washes in water, the amount of adsorbed CD-P remained exactly the same (Tab.1), indicating a good stability of the coating in aqueous solution.

These results were further confirmed by fluorescence spectroscopy studying the MIL-100 nanoMOFs interaction with rhodamine–labeled CD-P (CD-P-R). The amount of CD-P-R interacting with the nanoparticles after 1h of incubation, of 15.6 ± 3.2 wt%, was in good agreement with the results of the elemental analysis. Moreover, the observed payloads corresponded to a Fe trimers : βCDP molar ratio of about 13.3, suggesting that only a partial amount of the nanoparticles matrix is involved in the interaction.

**Tab S1**: Kinetics of CD-P association to the MIL-100 nanoMOFs express in terms of payload (PL, wt%), adsorption efficiency (AE, %) and Fe trimers/βCDP molar ratio achieved after different incubation times.

| **Time (h)** | **PL^*^ (wt%)** | **AE (%)** | **Fe trimers/CD-P molar ratio** |
| --- | --- | --- | --- |
| **0.25** | 13.1 | 30 | 14.5 |
| **1** | 14.1 | 32.6 | 13.3 |
| **24h** | 17.3 | 41.8 | 10.4 |
| **24h +**  **3 washes in water** | 17.3 | 41.8 | 10.4 |

*
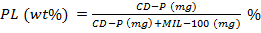


**II.2 MIL-100 nanoMOFs modification with CD-P: coating localization**

An “ideal” coating should allow an effective nanoMOFs functionalization without affecting their porosity and thus concerning only the nanoparticles external surface.

X ray photoelectron spectroscopy (XPS) was used to clearly ascertain the presence of CD-P within the nanoMOF top layers (~5-10 nm) and to determine the quantitative % atomic surface composition. In the case of the carbon C1s core level, the changes in the energy distribution of emitted photoelectrons allows a clear diagnostic of the MOF coating. Evolution of peak envelopes are “fingerprints” enabling to detect each component contribution: i) MIL-100(Fe) carbon skeleton (284.8 and 289 eV, assigned to C-C or to C-OOH, respectively) and ii) CD-P (with a main contribution at 286.3 eV) (see Fig. S1). So the additional characteristic peak contribution attributed to CD-P coating was detected through its specific shoulder together with the two main peaks of nanoMOFs. Note that the relative intensities of each feature are rather constant indicating a specific and reproducible thin CD-P coverage of MIL-100 external network. Further quantification of each constitutive element (C, O, P, Fe, Na and Cl) (table S2) indicated that P was not present in uncoated nanoMOFs, against 2.2 % atomic P for the coated ones. P signal is a demonstrating probe of the CD-P coverage. Its limited and reproducible amount is a second proof of a regular CD-P coverage onto MIL-100. Both on CD-P or MIL-100 CD-P covered samples only one P2p contribution is observed centered at 134eV typical of a PO_4_ presence. Another specific coverage parameter was the C/Fe atomic ratios which significantly increased from 7.4 to 12.1 before and after CD-P coating, in agreement with the presence of CD-P, also consistent with the evolutions of the O/Fe ratios. On the whole, using the characteristic %atomic ratio as the C1s 286.3 eV contribution or the unique P2p one at 134 eV, one can estimate that the XPS data comfort the assumption that the external surface of the nanoMOFs contains one CD-P molecule *per* iron trimer (SI) without penetration inside the porous network. Interestingly, despite the use of a Na salt of CD-P (Na/P ~ 1), the outer surface contained undetectable amounts of Na. This indicates that the Na^+^/phosphate pair has been replaced by stronger phosphate-iron(III) coordination, with probably most of the 3-4 P-O groups *per* CD-P interacting, providing thus a cooperative anchoring effect of the CD-P coating. Interestingly, the use of CD without phosphate groups did not lead to any successful grafting (Table S2, Fig. S1).

**Tab. S2:** Elemental composition of the MIL-100 nanoMOFs surface before and after incubation with CD-P as determined by XPS (At% = atomic %).

|  | **At%** | | | | | | |
| --- | --- | --- | --- | --- | --- | --- | --- |
| **Sample** | **P** | **Cl** | **C** | **N** | **O** | **Fe** | **Na** |
| MOF | 0 | 0.88 | 56.88 | 0 | 34.6 | 7.64 | 0 |
| MOF(CD-P) | 2.23 | 0.03 | 54.11 | 0 | 39.14 | 4.48 | 0 |
| CD-P | 7.67 | 0 | 39.71 | 0.43 | 44.42 | 0 | 7.77 |


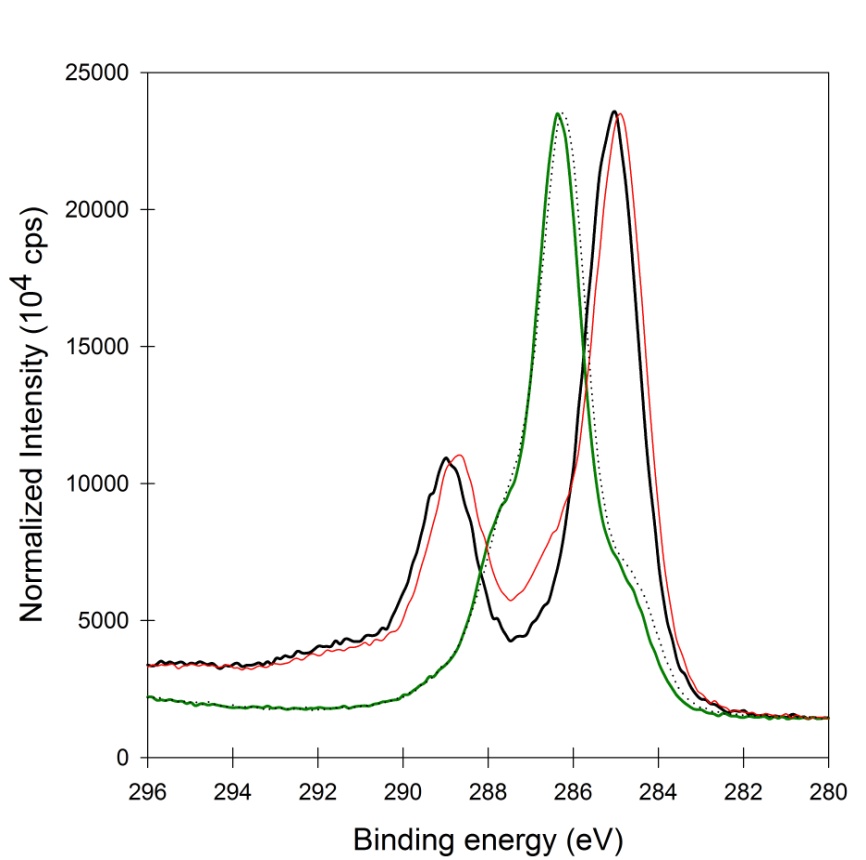


**Fig. S1:** C1s binding energy spectra obtained by XPS for nanoMOFs (black), CD-P (green) and CDP-modified nanoMOFs (red).

**II.3 MIL-100 nanoMOFs modification with PEG-amine: coating localization**

MIL-100 nanoMOFs were incubated 3h with a PEG-amine aqueous solution containing low or high doses of PEG. At the end of the incubation the nanoparticles were recovered by centrifugation, washed in water, in order to remove the excess of coating molecules not associated to the matrix and analyzed by XPS. The results are gathered in Table 3.

Traces of N, an atom exclusively belonging to PEG chains, were found on the nanoparticles surface and the C/Fe atomic ratio dramatically increased (7.4 and 12.8 in uncoated and Meo-PEG-NH_2_ modified MIL-100 nanoMOFs, respectively). The results confirm the effective presence of PEG chains at the nanoparticles surface.

**Tab. 3:** Elemental composition of the MIL-100 nanoMOFs surface before and after incubation with PEG-amino aqueous solution as determined by XPS (At% = atomic %).

|  | **At%** | | | | | |
| --- | --- | --- | --- | --- | --- | --- |
| **Sample** | **Cl** | **C** | **N** | **O** | **Fe** | **Na** |
| **MOF** | **0.88** | **56.88** | **0** | **34.6** | **7.64** | **0** |
| **MOF(PEG)** | **0.3** | **60.3** | **0.4** | **34.28** | **4.71** | **0** |
| **PEG** | **0** | **68.92** | **2.7** | **28.38** | **0** | **0** |

.

**II.4 MIL-100 nanoMOFs modification with CD-P: FT-IR analyses**


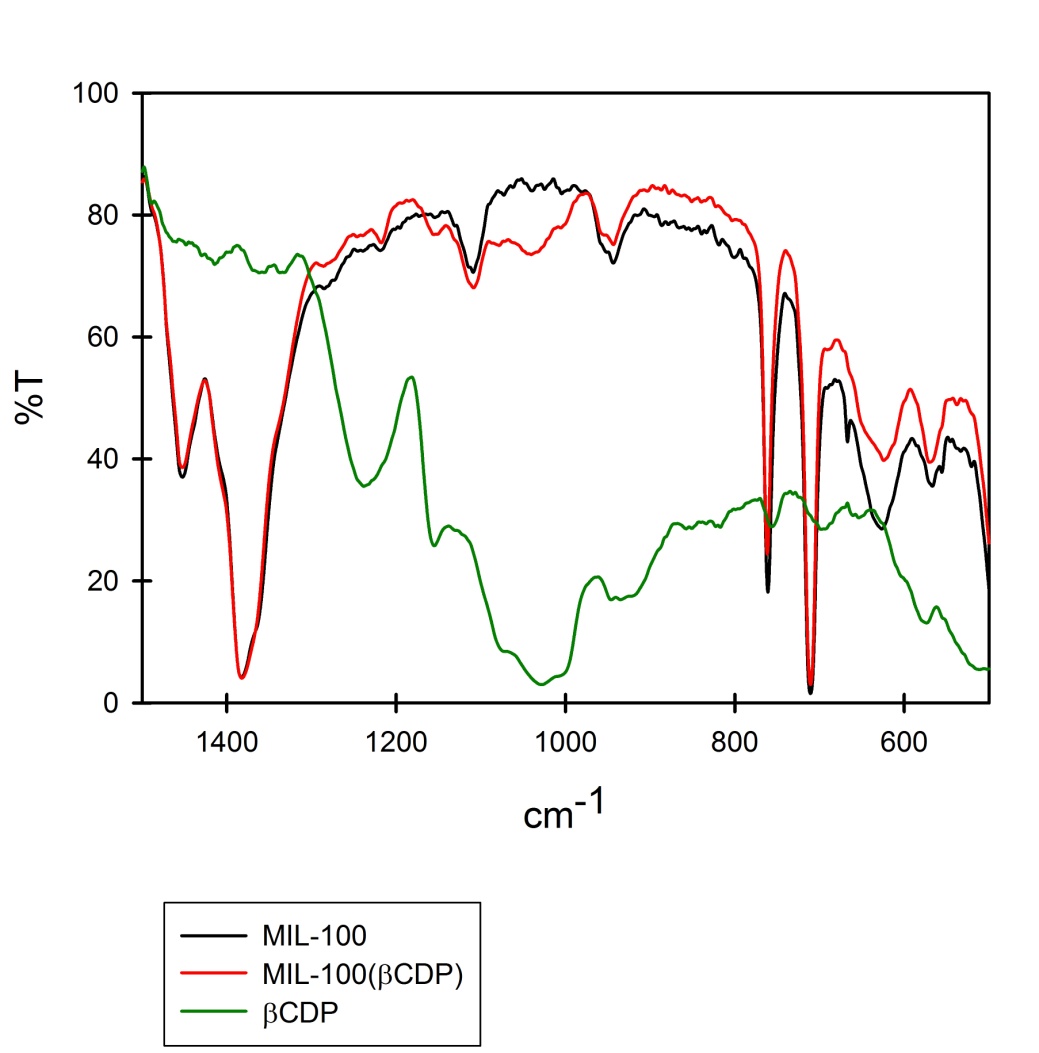


**Fig. S2:** FT-IR patterns of MIL-100 nanoMOF before (black line) and after (red line) modification with CD-P and CD-P powder sample (green line).

Coated nanoMOFs present both peaks of CD and MOFs.

**II.5 MIL-100 nanoMOFs modification with CD-P: solid-state NMR analyses**


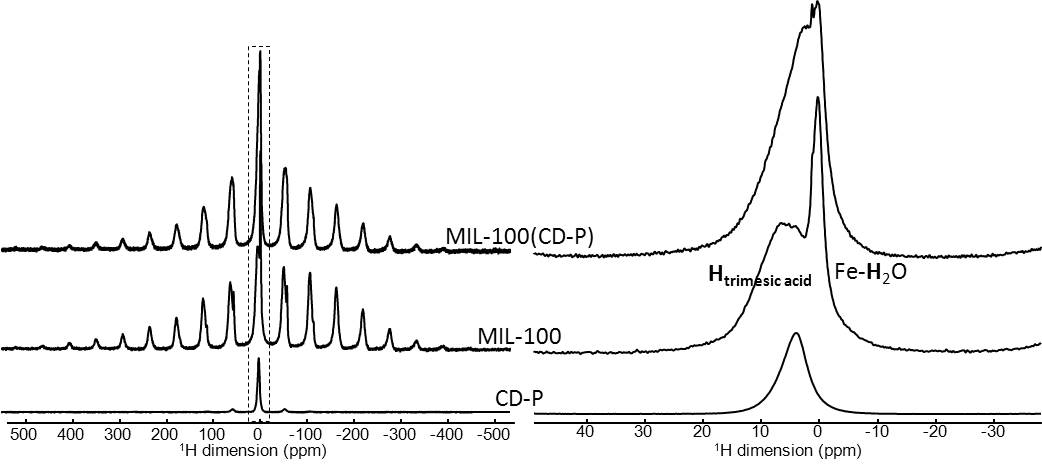


**Fig. S3:** Solid-state MAS (27 kHz) ^1^H NMR spectra of CD-P, MIL-100 nanoMOF and MIL-100(CD-P). The full spectra are shown on the left. The large number of spinning sidebands is du to the presence of the Fe paramagnetic center. Expansions of the isotropic positions are shown on the right.

The ^1^H NMR spectrum of the nanoMOF after incubation with CD-P still contains the main features of the empty MIL-100, indicating that the structure of the MOF was not modified by the coating process.

The upper spectrum contains, in addition to the ^1^H resonances from the MIL-100 nanoMOF (protons from the trimesic acid and protons from the water molecules bonded to the Fe ions), the signal of CD-P, confirming its attachment to the nanoMOFs. In this spectrum, the large spinning sideband manifold of the CD-P in the nanoMOF and the lower amount of Fe-H_2_O signal further confirm the close proximity between the CD-P and the paramagnetic center.

**II.6 MIL-100 nanoMOFs modification with CD-P: shell stability in physiological simulated conditions**


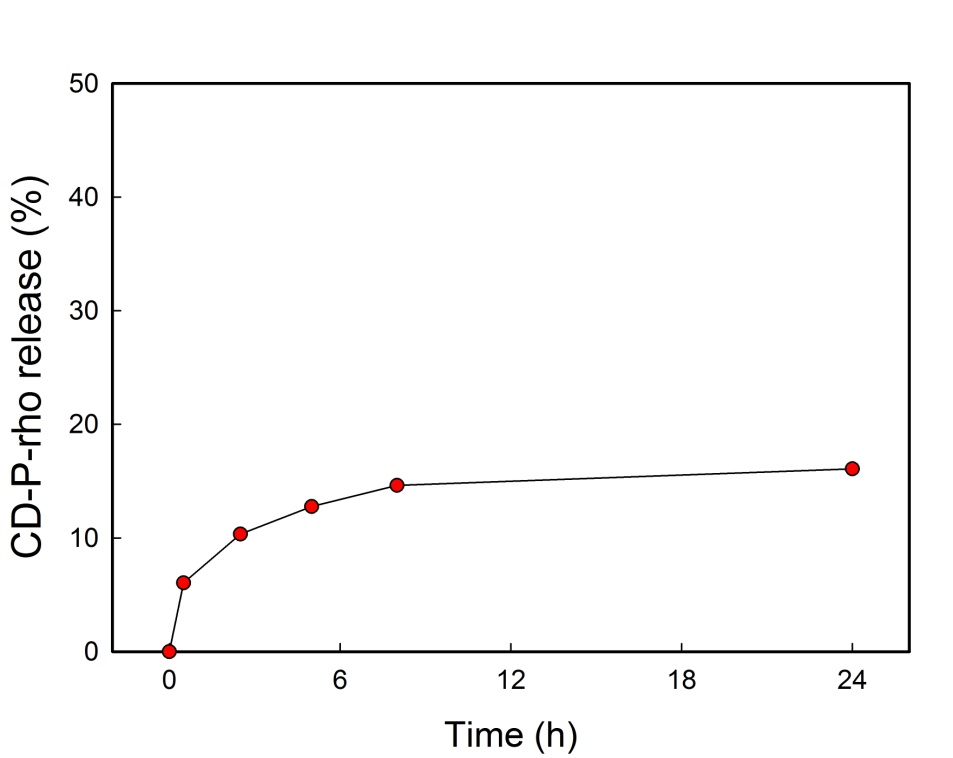


**Fig. S4:** Kinetics of rhodamine-labeled CD-P (CD-P-R) detachment from the MIL-100 nanoMOFs surface in RPMI supplemented with 10% FBS, at 37°C.

**II.7 Surface modification with CD-P of Aluminium trimesate nanoMOFs (MIL-100-Al)**

Aluminium trimesate nanoMOFs (MIL-100-Al) were synthesized and modified with a CD-P-R aqueous solution as reported in the experimental section.

At the end of the incubation, the amount of CD-P-R attached on the nanoparticles surface was to be 29.6(±0,6) wt%, as determined by spectrofluorimetry. The so-coated MIL-100-Al nanoMOFs were further resuspended in PBS at 37°C at a final nanoparticles concentration of 2mg/mL and the CD-P-R detecahment was monitored over time by spectrofluorimetry. As shown in Fig. S5, the CD-P-R-based coating is provided with a good stability in phosphate buffer with the 26.5(±4) % 37.1(±3.5) % of liberartion after 6 and 24h, respectively.

These results indicate that our strategy of nanoMOF surface modification can be extended to other nanomaterials built using different metals.

**
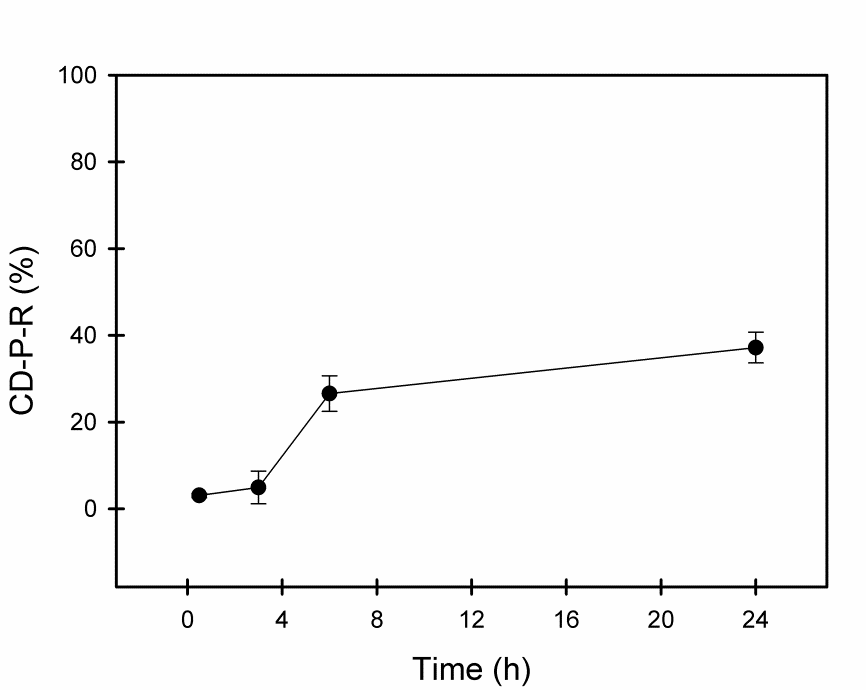
**

**Fig. S5:** Kinetics of rhodamine-labeled CD-P (CD-P-R) detachment from Aluminium trimesate nanoMOFs (MIL-100-Al) in PBS, at 37°C.


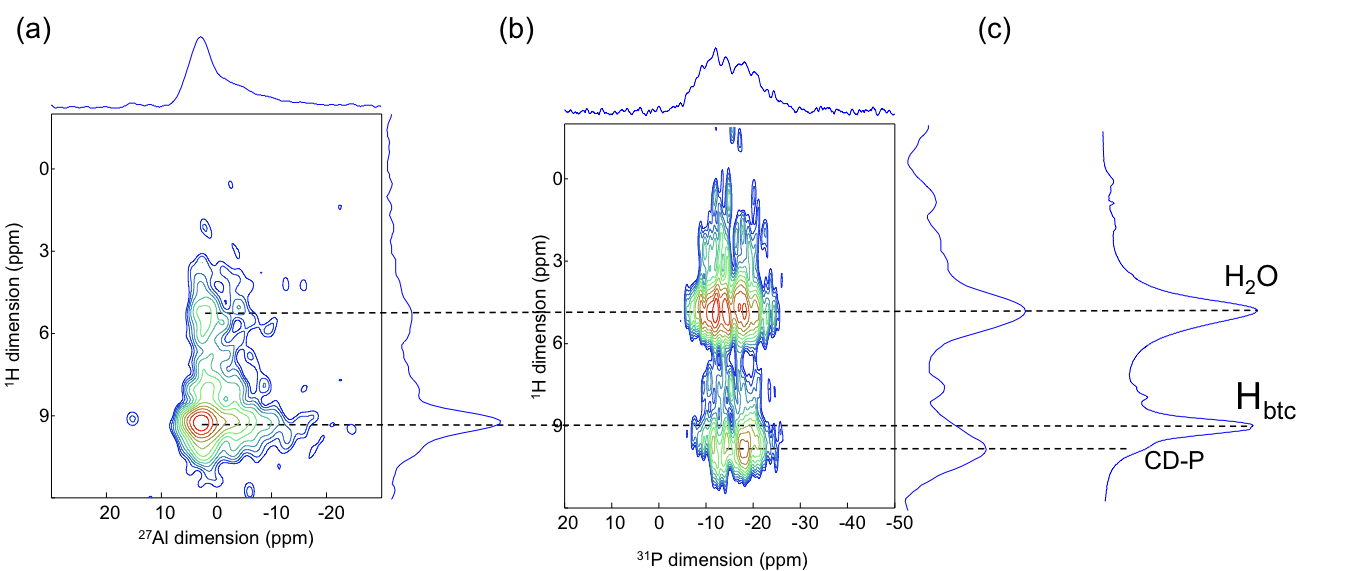


**Fig. S6.** (a) ^1^H→^27^Al and (b) ^1^H→^31^P 2D CP-HETCOR NMR spectra of MIL-100(Al) coated with CD-P. Top spectra are the full projections on the ^27^Al(^31^P) and ^1^H dimensions, respectively. In (c) is shown the ^1^H MAS NMR spectrum

The presence of CD-P in the coated MIL-100(Al) nanoMOFs is confirmed by the signal on the solid-state ^31^P NMR spectrum (top spectrum in Fig. S6b).

The ^27^Al-^1^H 2D spectrum (Fig. S6a) shows the two proton sites of the host nanoMOF MIL-100(Al) (*i.e.*, the water molecules from the Al clusters and the protons from the btc linkers).

The ^1^H→^31^P 2D NMR spectrum (Fig. S6b) contains correlation peaks between the phosphorus atoms of the CD-P and the protons of the water molecules, while there is no cross-peaks between these P atoms and the protons from the trimesic acid. This clearly confirms the preferential interactions of the CD-P with the Al cluster through the phosphate groups, and indicates that there is no CD-P in the pores.

**II.8 *In vitro* cytotoxic profile of uncoated and CD-P-modified MIL-100 nanoMOFs**


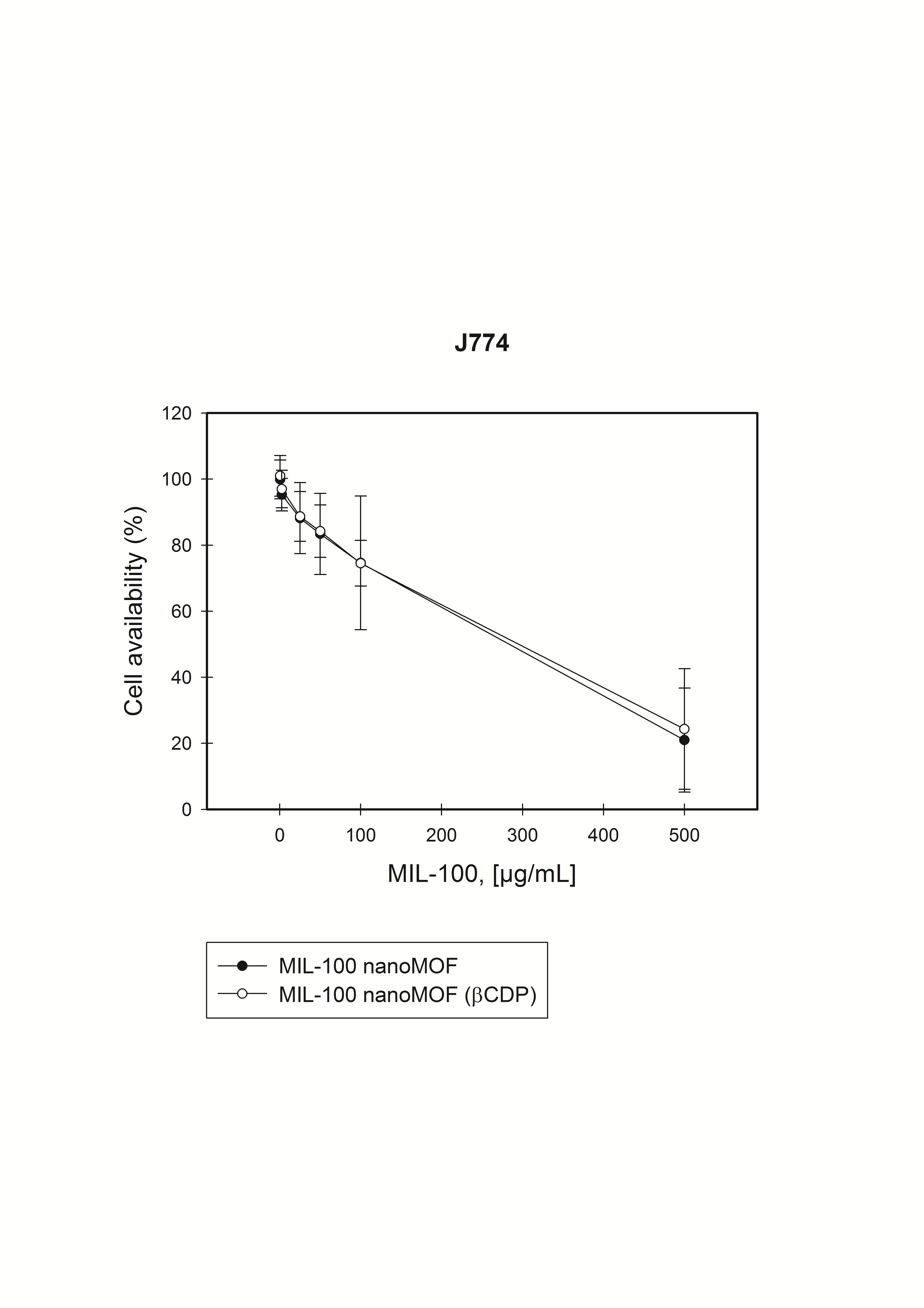

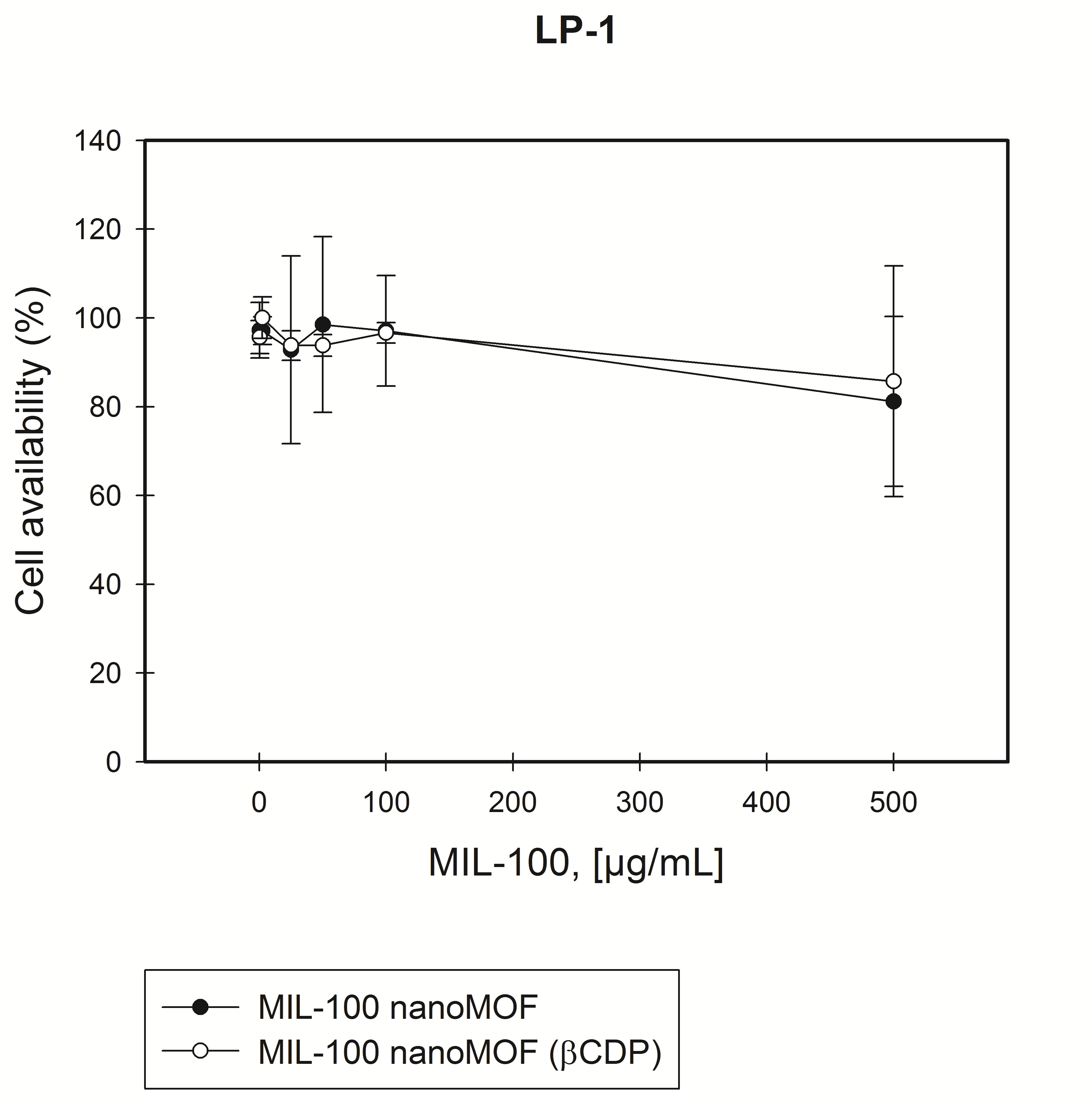

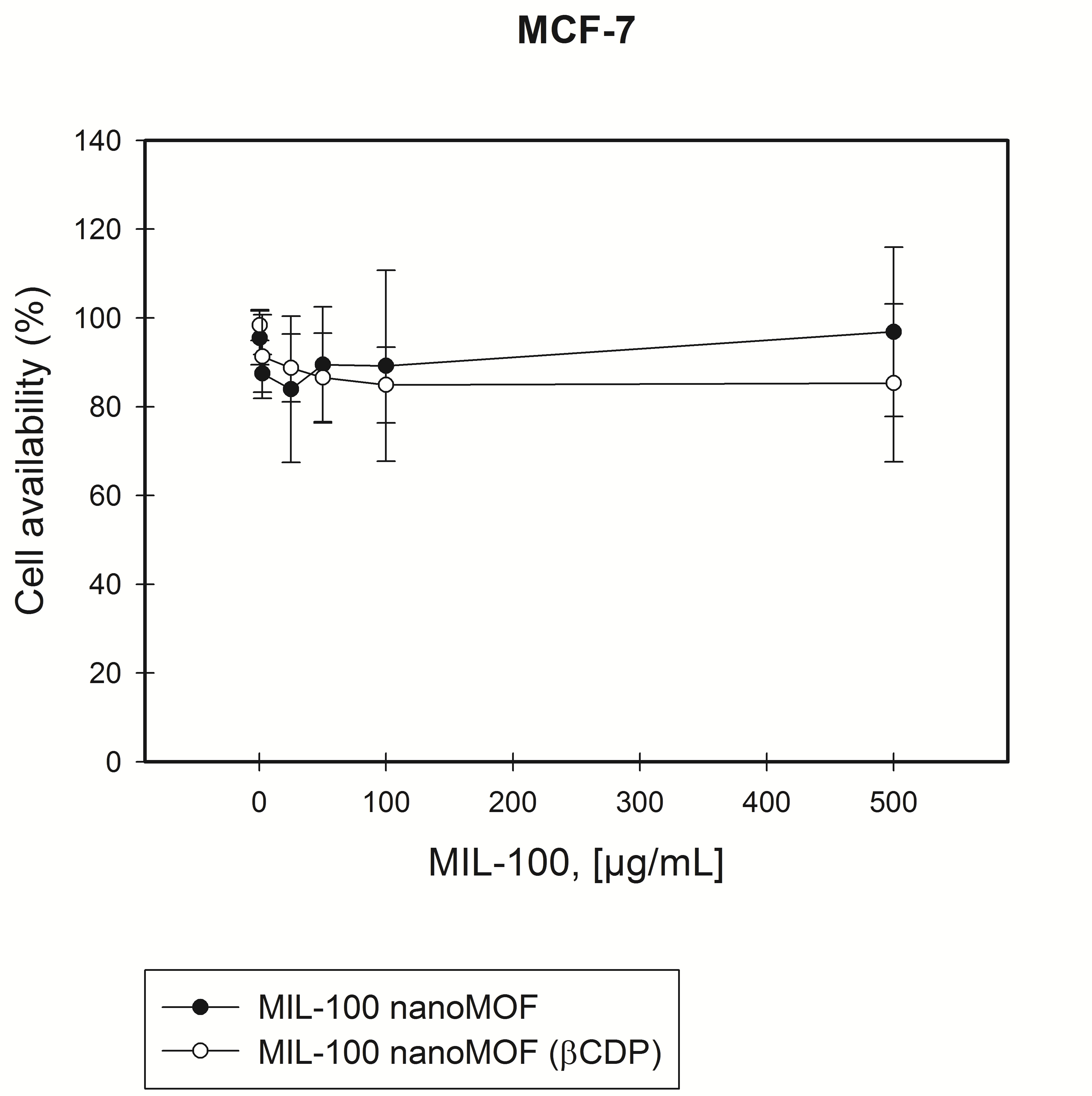


**Fig. S7:** Cell survival (%) of J774, LP1 and MCF-7 cell lines after 48h of incubation with different uncoated (black dot) and CD-P-modified MIL-100 nanoMOF as analyzed by MTT assay.

**II.9 Confocal images of a MOF particle coated with rhodamine-labelled CD-P**

***
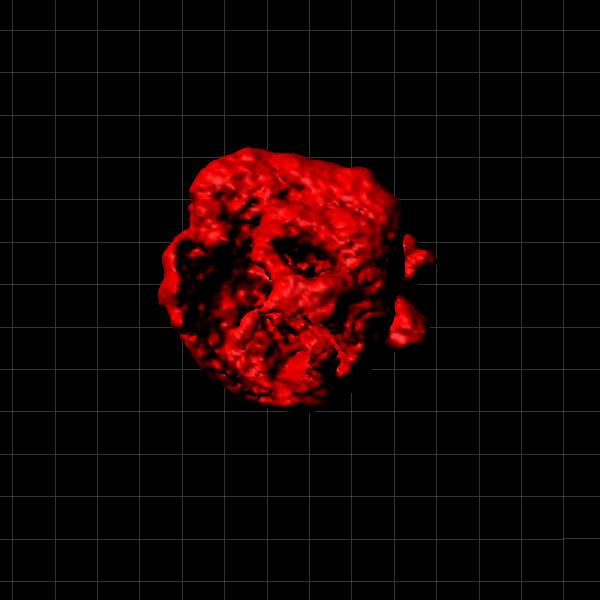

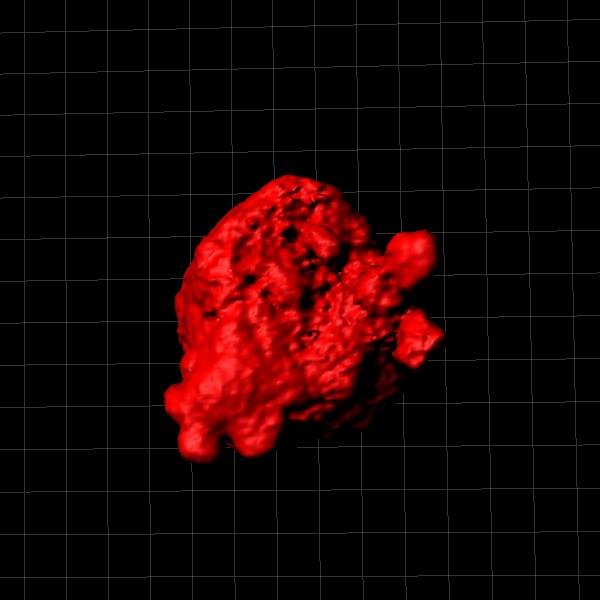
***

**a**

**b**

***
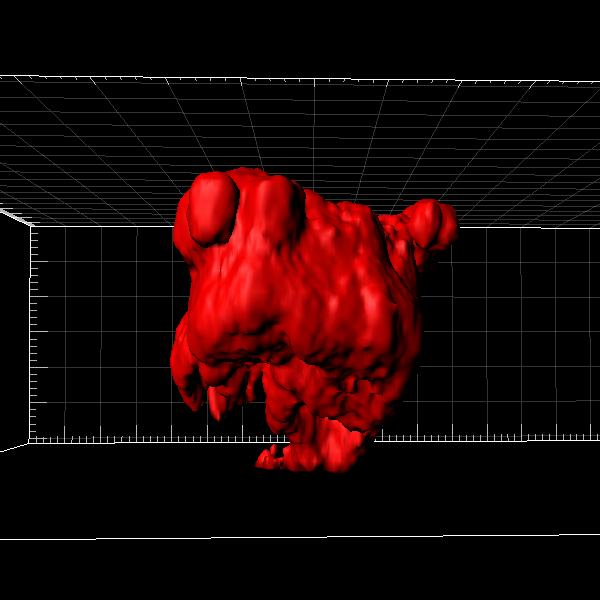

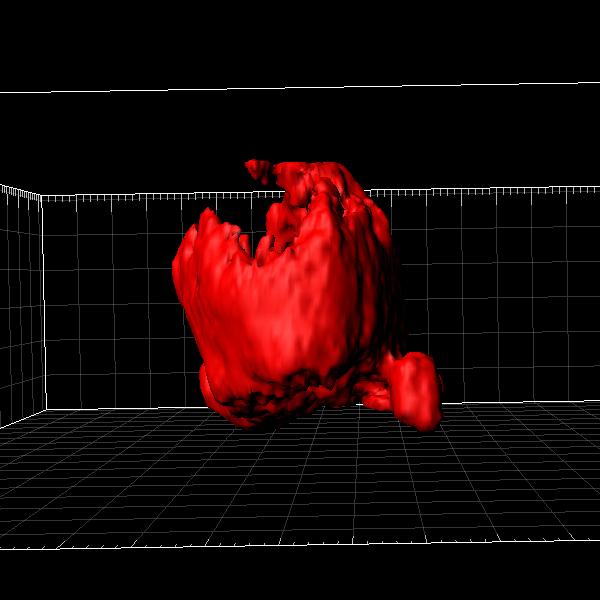
***

**c**

**d**

**Fig. S8: Reconstituted envelope of a MOF crystal coated with rhodamine-labelled CD-P.** Views from top (a) bottom (b) left (c) and right (d). Bar represents 10 μm.

Fig. S9 is a typical image of orthogonal projections of a particle inside a J774 macrophage showing that the particles penetrated within the cell with their rhodaminylated coating.


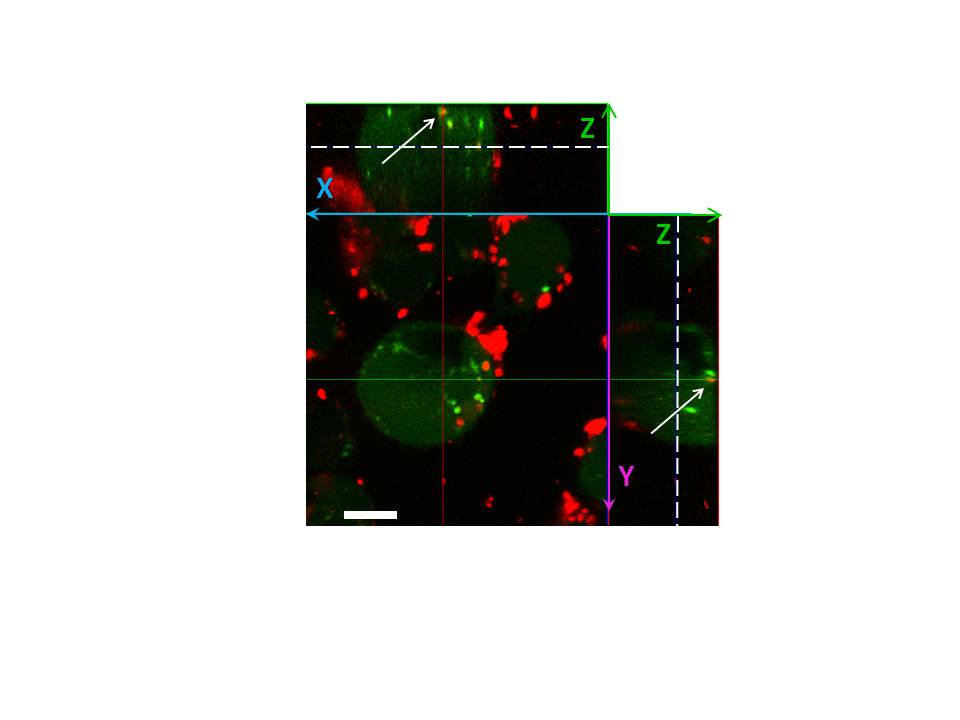


**Fig. S9:** Medial x-y plane and orthogonal x-z and y-z projections of J774 macrophages containing CD-P coated MOFs obtained by confocal microscopy. Cells were stained in green with calcein, whereas the red signal comes from rhodamin-labelled CD-P-coated MOFs. Cell nucleus appears in black. Bar represents 5 μm.

**II.10 Functionalization of CD-P coated nanoMOFs with Ad-PEG**

A strategy to functionalize the surface of nanoMOFs with PEG has been set up, based on the formation of inclusion complexes between an adamantyl (Ad) endgroup grafted on PEG (PEG-Ad) and β-CD-P cage molecules, which were shown previously to firmly adhere to the nanoMOF surfaces.

Indeed, Ad groups are known to be included and held strongly in β-CD, resulting in high association constants of their derivatives, reaching 10^5^ M^-1^. ^1-4^ As a result, inclusion complexes between Ad and β-CD are known to exhibit excellent stabilities in various media^5^. Such complexes were used in many fields, for example, Ad-modified contrast agents based on Gd^3+^ chelates were firmly entrapped in CDs in nanogels, opening a field of applications in magnetic resonance imaging.^6^

PEG-Ad has successfully been used to functionalize the surface of CD-based assemblies containing nucleic acids.^7^ These are the first siRNA delivery devices studied in humans. The PEG surface modification obtained by this way was efficient to confer *in vivo* long blood circulation times. Furthermore, transferrin (Tf), a targeting ligand, was grafted to one PEG chain end and Ad to the other, to obtain Ad-PEG-Tf which could be similarly used to modify the surface of the CD-based assemblies. The Tf ligand improved the uptake of the assemblies by tumor cells. Based on these results, the nanoassemblies named CALAA-01, made of cationic CD polymers loaded with siRNA against ribonucleotide reductase subunit 2, modified with Ad-PEG and Ad-PEG-Tf were used in May 2008 to treat patients in phase I clinical trials.^7^

In this study, we have first prepared β-CD-P: Ad-PEG inclusion complexes by mixing overnight at room temperature two aqueous solutions of these compounds, at a molar ratio of 2/1 (CD/Ad). Ad-PEG was synthesized as previously described^8^ by reacting O-(2-Aminoethyl)-O’-methylpolyethylene glycol (mPEG-NH_2_ MW 2,000 g/mol, Sigma–Aldrich) and 1-adamantyl isocyanate. 1-adamantyl is named here Ad.

The β-CD-P: Ad-PEG inclusion complexes were then associated to the nanoMOFs, following the same incubation procedure as described in I.5 (Fig. S10). β-CD-P: Ad-PEG coated nanoMOFs were characterized as described previously (sections I.5-I.8). The size of the nanoMOFs was not affected by the coating procedure, as reported also in the main text in the case of CD-P coatings.


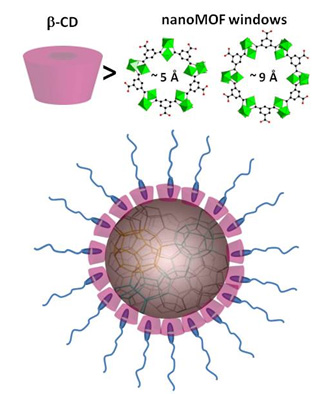


**Fig. S10: Schematic representation of the PEG surface modification.** Upper panel: β-CDs are too large to cross the MOF pentagonal and hexagonal windows of around 5 and 9 Å, respectively. Lower panel: β-CD-P: Ad-PEG complexes are first formed, then associated to the nanoMOF surface by complexation between the P groups and the iron sites available on the MOF surface.

Isothermal titration calorimetry (ITC) is one of the key method used here, sensitive to study interactions in complex systems and widely used to investigate the inclusion complexes between CDs and invited molecules. One advantage of ITC in studying complex formation is the possibility to directly estimate the binding enthalpy (Δ*H*), which in conjunction with the estimated association constant (K), allows the calculation of the free energy (Δ*G*) and entropy of binding (Δ*S*). In the present study, ITC was used as a powerful tool enabling to assess all these parameters. The inclusion compounds studied were: as hosts, β-CD and β-CD-P, and as guests, Ad and Ad-PEG (Fig. S11). A typical example of the exothermic interactions arising in all the studied cases is given in the upper panel in Fig. S10. Exothermic heat flows are released upon successive injection of 10 μL aliquots of Ad-PEG into β-CD-P solutions. The bottom panel in Fig. S10 shows integrated heat data, giving a differential binding curve which was fit to a standard single-site binding model. The calculated association constants K and the thermodynamic parameters characterizing the four host-guest systems are presented in Table 4.

First, it can be observed that the interaction between Ad and β-CDs is strong as characterized by an association constant of 1.6 10^4^ M^-1^. This value is in perfect agreement with previous data^5^ reporting association constants of 1.7 10^4^ M^-1^.

**Tab. 4: Association constants and thermodynamic parameters characterizing the interaction between Ad and Ad-PEG and β-CD bearing or not phosphate groups.**

| **Host** | **Guest** | **K (M^-1^)** | **∆H**  **(kJ.mol^-1^)** | **∆G**  **(kJ.mol^-1^)** | **T∆S (kJ.mol^-1^)** |
| --- | --- | --- | --- | --- | --- |
| **β-CD** | **Ad** | 16,000 | -14.8 | -23.9 | 9.2 |
| **β-CD-P** | **Ad** | 72,800 | -14.9 | -27.7 | 12.7 |
| **β-CD** | **Ad-PEG** | 33,400 | -21.5 | -25.8 | 4.4 |
| **β-CD-P** | **Ad-PEG** | 30,000 | -14.6 | -25.4 | 10.9 |

Table 4 shows that the interactions of Ad and Ad-PEG with both β-CD and β-CD-P were exclusively exothermic phenomena (Δ*H*< 0) with positive entropic contribution (Δ*S* > 0) and mostly enthalpy driven (|Δ*H*| > |*T*Δ*S*|). Because large enthalpic gains were observed, it is suggested that the interactions of Ad and Ad-PEG with both β-CD and β-CD-P are predominantly mediated by the formation of van der Waals-type bonds as described in the literature.^9^

*T*Δ*S* increased from 9.2 to 12.7 kJ.mol^-1^ in the case of Ad interactions with β-CD and β-CD-P, respectively. The same trend was observed for Ad-PEG interacting with β-CD and β-CD-P, where *T*Δ*S* increased from 4.4 to 10.9 kJ.mol^-1^. The entropy increase might be attributed to an enhancement of the degree of freedom of Ad upon complexation with β-CD-P as compared to β-CD.

Because of the grafted P groups, the environment of the CD cavity is more hydrophilic in β-CD-P than in β-CD. One could thus expect that the reorganization of surface/cavity neighbouring water molecules that are released upon guest inclusion is higher in the case of β-CD-P than in β-CD, resulting in more positive entropy changes. Furthermore, the desolvation upon guest inclusion and the induced dehydration of the hydroxyl groups in β-CD-P could be responsible for an entropic gain.

Among the four host:guest interactions studied (Table 4) the highest affinity constant was found for the Ad: β-CD-P interaction. The exact role of the P groups in enhancing the interaction as compared to the Ad: β-CD one (K around 1.6 10^4^ M^-1^) is not fully understood, but several parameters are believed to pay a role in this interaction, such as: i) a hydrophilicity increase of β-CD-P as compared to β-CD and ii a change in flexibility and geometry of the CD cavity following P grafting.

Interestingly, Ad-PEG interacted strongly with both β-CD and β-CD-P leading to K in the range 3-3.3 10^4^ M^-1^.

**Fig. S11: ITC thermograms characterizing the interactions between β-CD and β-CD-P as hosts and Ad and Ad-PEG as guests.** Upper panel: a typical example of exothermic heat flows released upon successive injection of 10 μL aliquots of Ad-PEG (2.5 mM in Ad) into β-CD-P solutions (0.25 mM in β-CD). Bottom panel: Integrated heat data, giving a differential binding curve which was fit to a standard single-site binding model. Black: Ad (2.5 mM) titrating β-CD (0.25 mM); Pink: Ad (2.5 mM) titrating β-CD-P (0.25 mM in β-CD); Green: Ad-PEG (2.5 mM in Ad) titrating β-CD (0.25 mM) and Red: Ad-PEG (2.5 mM in Ad) titrating β-CD-P (0.25 mM in β-CD).

Finally, the interaction between β-CD-P: Ad-PEG complexes and nanoMOFs has been studied and compared to the one between PEG (non functionalized) and nanoMOFs (Fig. S12).

Fig. S12 reveals that the interaction between PEG and the nanoMOFs is low, supporting our hypothesis of a rather poor affinity between these materials. Indeed, Δ*H* characterizing the PEG:nanoMOF interaction (16.5 kJ.mol^-1^) is almost five times lower than Δ*H* characterizing the interaction between β-CD-P: Ad-PEG complexes and nanoMOFs (79.9 kJ.mol^-1^).

In conclusion, ITC data show that Ad-PEG strongly interacts with β-CD-P. The resulting inclusion complexes Ad-PEG: β-CD-P bind to the nanoMOF in a very similar way as β-CD-P. ITC titration curves show at each injection, endothermic phenomena, corresponding to the formation of coordination bonds (P-Fe) followed by matrix dehydration, as discussed previously in the main text. These data open up new possibilities of surface functionalization following a Lego-type assembly of Ad-PEG derivatives bearing or not ligands, as in previous studies undertaken with CALAA-1, a β-CD-based delivery system which was considered for clinical trials.

The interest of nanoMOF surface modification with Ad-PEG has been shown in our preliminary studies of complement activation. Coating nanoMOFs with Ad-PEG: β-CD-P instead of β-CD-P enabled to reduce by two fold complement activation. Before proceeding to the final *in vivo* proof of concept studies, optimization of the coating in terms of PEG surface density is necessary, as described in the literature.^10^ These studies are now underway.

**Fig. S12:** Thermograms obtained by ITC as the result of the interaction between: i) β-CD-P: Ad-PEG complexes (1.95 mM in PEG) and nanoMOFs (0.75 mM in iron sites) (black) and ii) PEG (1.95 mM) and nanoMOFs (0.75 mM in iron sites) (red).

**Supplementary References**

1. W.C. Cromwell, K. Bystrom, M.R. Eftink, Cyclodextrin–adamantanecarboxylate inclusion complexes: studies of the variation in cavity size, *J. Phys. Chem*., 1985, **89**, 326-332.
2. M.R. Eftink, M.L. Andy, K. Bystrom, H.D. Perlmutter, D.S. Kristol, Cyclodextrin inclusion complexes: studies of the variation in the size of alicyclic guests, *J. Am. Chem. Soc*., 1989, **111**, 6765-6772.
3. F.P. Charbonnier, S. Penadés, A straightforward synthesis of 1-adamantylmethyl glycosides, and their binding to cyclodextrins, *Eur. J. Org. Chem*., 2004, **17**, 3650-3656.
4. V.H. Tellini, A. Jover, L. Galantini, F. Meijidea, J.V. Tato, . Crystal structure of the supramolecular linear polymer formed by the self-assembly of mono-6-deoxy-6-adamantylamide-beta-cyclodextrin, *Acta Crystallogr. B*, 2004, **60**, 204-210.
5. M. Othman, K. Bouchemal, P. Couvreur, R. Gref, Microcalorimetric investigation on the formation of supramolecular nanoassemblies of associative polymers loaded with gadolinium chelate derivatives, *Int. J.Pharm*., 2009, **379**, 218-225.
6. E. Battistini, E. Gianolio, R. Gref, P. Couvreur, S. Fuzerova, M. Othman, S. Aime, B. Badet, P. Durand, High relaxivity MRI contrast agents based on supramolecular assembly between a gadolinium chelate, a modified dextran and poly-β-cyclodextrin, *Chem. Europ. J.,* 2008, **14**, 4551-4561.
7. M.E. Davis, The first targeted delivery of siRNA in humans via a self-assembling, cyclodextrin polymer-based nanoparticle: from concept to clinic, *Mol. Pharm.*, 2009, **6**, 659-668.
8. A.M. Layre, G. Volet, V. Wintgens, C. Amiel, Associative Network Based on Cyclodextrin Polymer: A Model System for drug Delivery, *Biomacromolecules*, 2009, **10**, 3283-3289.
9. M.V. Rekharsky, Y. Inoue, *J. Am. Chem. Soc*., 2002, **124**, 813–826.
10. R. Gref et al., 'Stealth' corona-core nanoparticles surface modified by polyethylene glycol (PEG): influences of the corona (PEG chain length and surface density) and of the core composition on phagocytic uptake and plasma protein adsorption, *Colloids Surfaces B-Biointerfaces*, 2000, **18**, 301-313.
